# Loss of *Kmt2c*/*d* promotes gastric cancer and confers vulnerability to mTORC1 and PD-1 inhibition

**DOI:** 10.1172/JCI194462

**Published:** 2026-05-12

**Authors:** Naitao Wang, Dan Li, Tao Zhang, Mohini R. Pachai, Dana M. Schoeps, Yudi Bao, Woo Hyun Cho, Makhzuna N. Khudoynazarova, Kae Kristoff, Marion Liu, Laura Tang, Yelena Y. Janjigian, Ping Chi, Yu Chen

**Affiliations:** 1 Shenzhen University Medical School, Shenzhen, China.; 2 Human Oncology and Pathogenesis Program, Memorial Sloan Kettering Cancer Center, New York, New York, USA.; 3 Peking University People’s Hospital, Beijing, China.; 4 Department of Surgery,; 5 Department of Pathology, and; 6 Department of Medicine, Memorial Sloan Kettering Cancer Center, New York, New York, USA.; 7 Department of Medicine, Weill Cornell Medical College, New York, New York, USA.

**Keywords:** Gastroenterology, Genetics, Oncology, Epigenetics, Gastric cancer, Mouse models

## Abstract

Based on the observation that loss-of-function mutations of *KMT2C* and *KMT2D* (*KMT2C/D*) are enriched and co-occur in gastric adenocarcinoma, we developed genetically engineered mouse models (GEMMs) to conditionally knock out *Kmt2c* and *Kmt2d* in gastric epithelial cells. We observed that *Kmt2c/d* loss led to nuclear dysplasia, cellular crowding, and expansion of cells with mixed gastric lineage markers. When combined with *Pten* deletion, *Kmt2c/d* loss drove rapid development of muscle-invasive gastric adenocarcinoma as early as 3 weeks after Cre-mediated gene deletion. The adenocarcinoma exhibited decreased expression of gastric lineage markers and increased expression of intestinal differentiation markers, phenocopying human intestinal-type gastric adenocarcinoma. Bioinformatic integration of single-cell RNA-seq of our GEMMs and human gastric cancer datasets showed coclustering of normal and of cancerous gastric epithelial cells. *Kmt2c/d* knockout in gastric epithelium reduced protein synthesis but upregulated transcription of ribosomal proteins, rendering the cells hypersensitive to mTOR complex 1 (mTORC1) inhibitors. Additionally, *Kmt2c/d* knockout increased MHC class I molecule expression and enhanced antigen presentation. Combination of mTORC1 inhibition and anti–programmed cell death 1 immunotherapy markedly suppressed tumor growth in immune-competent mice. Together, these findings reveal the role of *Kmt2c*/*d* loss in gastric cancer initiation and suggest potential therapeutic strategies for *KMT2C/D*-deficient gastric cancer.

## Abstract 2

We modeled gastric cancer progression with knockout of Kmt2c and Kmt2d that led to dysplasia and together with Pten knockout that led to invasive adenocarcinoma.

## Introduction

Gastric or stomach adenocarcinoma (STAD) is the fifth most common cancer and the fourth most common cause of cancer death worldwide (1). Gastric cancer exhibits histological and molecular heterogeneity, correlating with diverse genetic mutations and clinical outcomes (2). Based on molecular profiles, STAD can be classified into 4 major subtypes: microsatellite instability (MSI), Epstein-Barr virus–positive (EBV), genomically stable (GS), and chromatin instability (CIN) (3, 4). Histologically, MSI, EBV, and CIN tumors are enriched for intestinal subtype and GS tumors for diffuse subtype (5). Phosphatidylinositol-4,5-bisphosphate 3-kinase catalytic subunit alpha (*PIK3CA*) hotspot mutations are the most prevalent in EBV subtype, loss of cadherin 1 is the dominant mutation in GS subtype, and *TP53* loss-of-function (LOF) mutations are the leading alteration in CIN subtype. While dysfunction of the mismatch repair (MMR) system is the primary cause of hypermutagenesis in MSI subgroup, the drivers of cancer initiation in this subtype remain largely unexplored. In the MSI subgroup, mutations in the RAS and phosphatidylinositol-3-kinase (PI3K) pathway, and epigenetic modifiers, such as *KMT2D*, *ARID1A*, and *KMT2C*, represent top genetic alterations (3, 4). Recent studies using human stomach organoid and genetically engineered mouse models (GEMMs) have demonstrated that loss of *ARID1A* promotes cancer progression and impairs tumor differentiation (6, 7). However, the roles of *KMT2C* and *KMT2D* alterations in STAD remain unclear.

KMT2C and KMT2D are members of type 2 histone lysine methyltransferase (KMT2) family (8, 9). The primary catalytic function of KMT2C/D is to mediate mono- and di-methylation of histone 3 lysine 4 (H3K4me1 and H3K4me2) at active enhancers and a subset of CpG-low promoters (10, 11). Previous studies have demonstrated the tumor-promoting effects of *KMT2C/D* LOF mutations in lymphomas, urothelial cancer, lung cancer, and breast cancer (10, 12–16). In STAD, most *KMT2C/D* mutations are LOF mutations, such as frameshift and nonsense mutations that lead to protein truncations (17), suggesting that the subsequent reduced H3K4 methylation and dysregulated gene expression may contribute to tumorigenesis in gastric cancer as well. Consistently, inhibiting lysine-specific demethylase 1 (LSD1), one of the demethylases of H3K4 methylations, has been shown to suppress gastric cancer cell growth in cell culture and xenograft models (18). Since LSD1 inhibition increases H3K4 methylation, we hypothesize that loss of *KMT2C/D* and the consequent decrease of H3K4 methylation may promote cancer cell growth (18). To date, only a few studies have explored the molecular features and clinical implications of *KMT2C/D* mutations in the context of immunotherapy (19).

To characterize the functional consequences of *KMT2C/D* loss in gastric cancer, we developed GEMMs to conditionally knock out *Kmt2c*/*d* in gastric epithelial cells using *Tmprss2-CreER^T2^*. We investigated the histological and transcriptional changes and explored the underlying mechanisms and therapeutic opportunities.

## Results

### Co-occurrence of KMT2C and KMT2D LOF mutations and PI3K pathway alterations in STAD.

**Figure 1:**
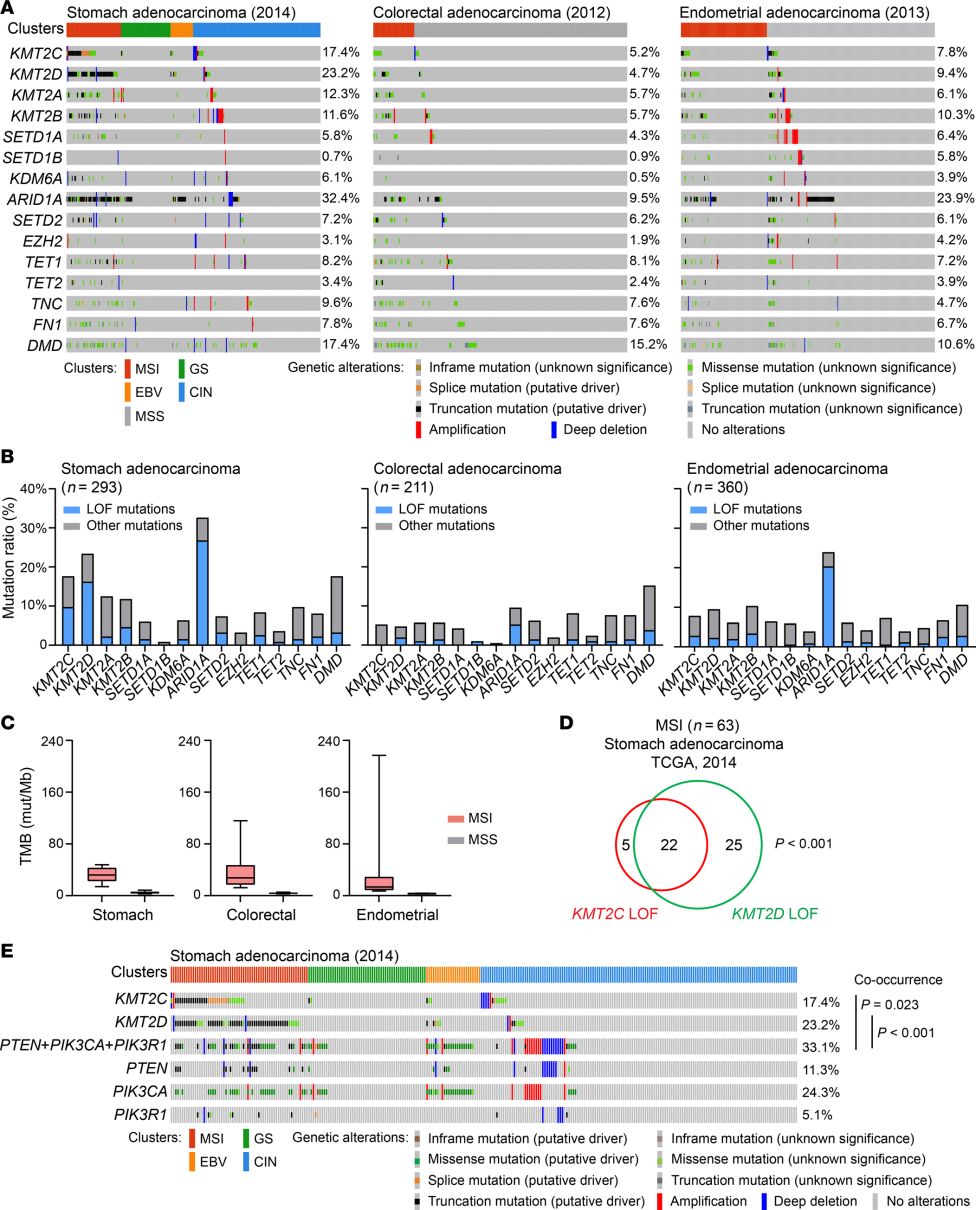
Co-occurrence of *KMT2C/D* LOF mutations and PI3K pathway alterations in STAD. (**A**) OncoPrint of selected chromatin modifying genes and several other large genes in TCGA datasets of STAD from 2014, colorectal adenocarcinoma from 2012, and endometrial adenocarcinoma from 2013. Samples in stomach adenocarcinoma were sorted as MSI, GS, EBV, and CIN groups. Samples in colorectal adenocarcinoma and endometrial adenocarcinoma were sorted as MSI and microsatellite stable (MSS). DMD, dystrophin. (**B**) Percentage of loss-of-function (LOF) mutations and other mutations in STAD, colorectal adenocarcinoma, and endometrial adenocarcinoma. Splice and nonsense mutations that lead to protein truncations were considered as LOF mutations. (**C**) Tumor mutational burden in MSI (hypermutated) and MSS (nonhypermutated) cancer samples. The center line represents the median, the box limits represent the upper and lower quartiles, and the minimum and maximum whiskers represent the 10th and 90th percentiles, respectively. (**D**) Venn diagram showing the overlap of *KMT2C* and *KMT2D* LOF mutations in MSI STAD samples. Statistical significance was determined using 2-tailed χ^2^ test. (**E**) OncoPrint of *KMT2C*, *KMT2D*, and mutations of PI3K signaling (including *PTEN*, *PIK3CA*, and *PIK3R1*). Mutual co-occurrences were observed between pooled mutations of PI3K signaling and *KMT2C* or *KMT2D*. Statistical significance was determined using 2-tailed χ^2^ test.

To examine the mutational landscape of *KMT2C* and *KMT2D*, we analyzed The Cancer Genome Atlas (TCGA) dataset, focusing on MSI-enriched cancer types, e.g., stomach, colorectal, and endometrial adenocarcinoma (3, 20, 21). In the original STAD TCGA dataset of 294 samples (3), *KMT2C* mutations were identified in 17.4% of samples, and *KMT2D* mutations were found in 23.2% of samples, with a substantial enrichment in the MSI subgroup (Figure 1A and Supplemental Figure 1A; supplemental material available online with this article; https://doi.org/10.1172/JCI194462DS1). Among the additional 145 samples included in the PanCancer TCGA (22), *KMT2C* and *KMT2D* mutations were similarly enriched in the MSI-high subgroup (Supplemental Figure 1, B and C). Most *KMT2C* and *KMT2D* mutations were bona fide LOF (i.e., truncation and splice site mutations) instead of missense or in-frame mutations of unknown function, suggesting that *KMT2C/D* LOF mutations may be positively selected in cancer progression (Figure 1B). Further transcriptional analysis revealed that downregulation of *KMT2C* or *KMT2D* did not substantially contribute to their dysfunction in gastric cancer (Supplemental Figure 1, D and E). Given the high mutational burden in the MSI subgroup because of dysfunction of the MMR system, we analyzed the mutational landscape of other epigenetic modifiers and several comparably large genes. In STAD, we observed that high percentages of *ARID1A* (82.1%), *KMT2D* (69.1%), and *KMT2C* (54.9%) mutations were LOF (Figure 1B). In contrast, the majority of mutations of other epigenetic modifiers were missense, and the LOF mutation rate of *DMD*, the largest known human gene, was only 17.6% (Figure 1B).

To determine whether the positive selection of *KMT2C/D* LOF mutations is specific to STAD, we analyzed their mutational profiles in colorectal and endometrial adenocarcinoma, 2 other cancer types with a high proportion of MSI tumors (20, 21). Notably, we observed positive enrichment of LOF mutations in *ARID1A* but not in *KMT2C* or *KMT2D* in these 2 cancer types (Figure 1B). The mutational rates of other large genes, such as *TNC*, *FN1*, and *DMD*, were comparable across these cancer types, and the mutational burdens in MSI samples were similar (Figure 1, B and C). Additionally, we observed the mutual co-occurrence of *KMT2C* and *KMT2D* LOF mutations in the MSI subgroup of STAD (Figure 1D). These data suggest that *KMT2C* and *KMT2D* LOF mutations may cooperatively contribute to tumorigenesis in STAD.

Dysregulation of the PI3K pathway (e.g., LOF mutations in phosphatase and tensin homolog [*PTEN*] and *PIK3R1* and gain-of-function mutations in *PIK3CA*) is common in gastric cancers (Figure 1E), particularly in the MSI and EBV subgroups in both TCGA and The Asian Cancer Research Group cohorts (3, 4). Moreover, *PTEN* inactivation has been shown to accelerate gastric cancer progression (23, 24). In the TCGA dataset, we observed a high frequency of PI3K pathway alterations, most commonly through *PTEN* LOF mutations and *PIK3CA* hotspot gain-of-function mutations in the MSI subgroup (Figure 1E). Additionally, there was a significant co-occurrence of *KMT2C/D* mutations with PI3K pathway alterations (Figure 1E), indicating a possible synergistic role in gastric cancer pathogenesis.

### Kmt2c/d knockout cooperates with Pten loss to induce muscle-invasive gastric cancer.

**Figure 2:**
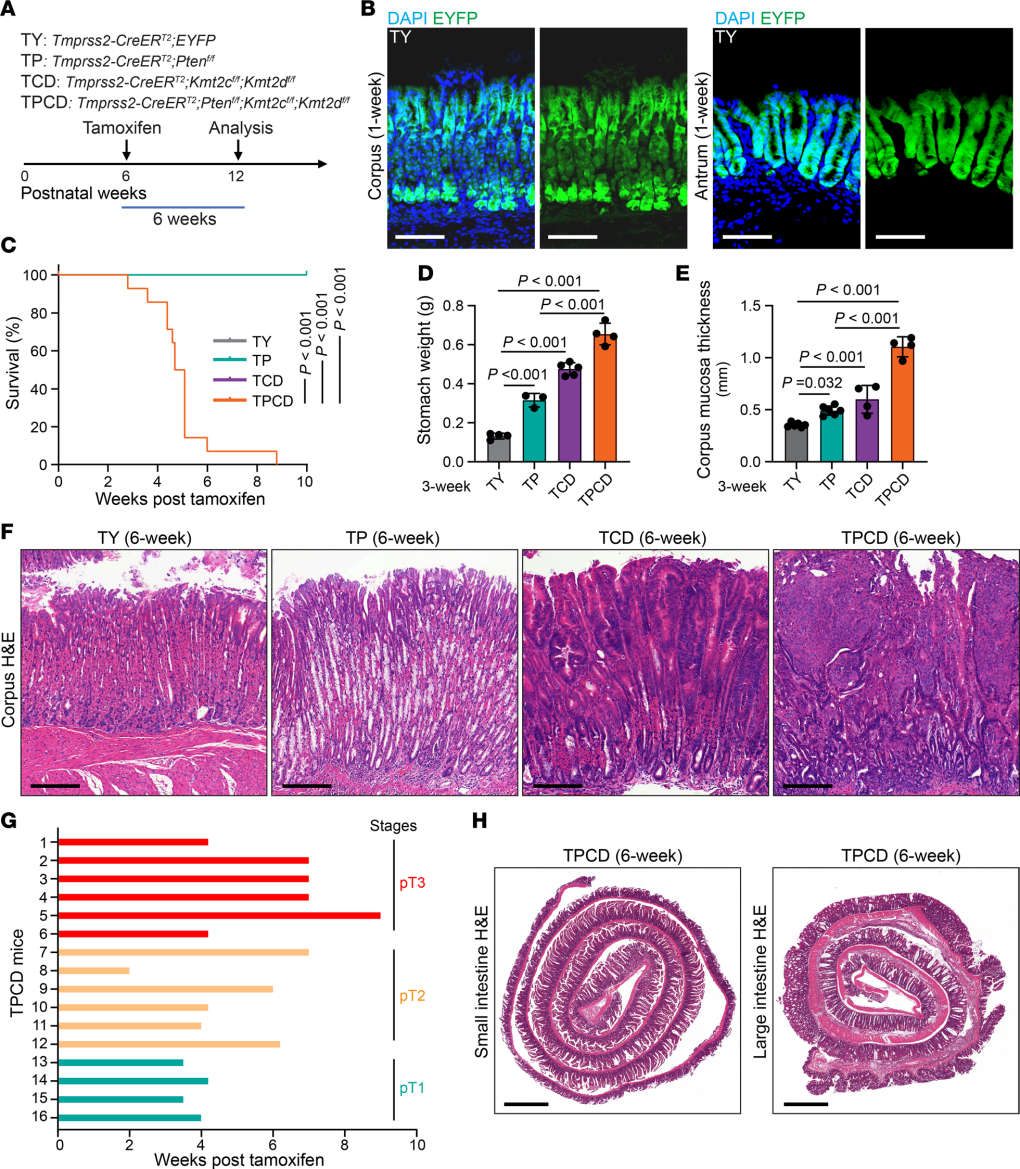
*Kmt2c/d* knockout cooperates with *Pten* loss to induce muscle-invasive gastric cancer. (**A**) Schematic of mouse models: 2 doses of tamoxifen (3 mg × 2) were injected intraperitoneally with a 48-hour interval. (**B**) Representative immunofluorescence (IF) staining of EYFP in stomach tissues from *Tmprss2-CreER^T2^ Rosa26-LSL-EYFP* mice. Nuclei were counterstained with DAPI. Scale bar, 200 μm. (**C**) Kaplan-Meier plots showing the survival of mice after gene knockout. TY (*n* = 10 mice), TP (*n* = 11 mice), TCD (*n* = 9 mice), TPCD (*n* = 14 mice). (**D**) Stomach weight of the indicated GEMM 6 weeks after tamoxifen injection. Data are presented as mean ± SD and analyzed using 2-way ANOVA followed by Tukey’s multiple comparisons test. TY (*n* = 4 mice), TP (*n* = 3 mice), TCD (*n* = 5 mice), TPCD (*n* = 4 mice). (**E**) Thickness in corpus mucosa measured by microscopy of H&E staining section. Each dot represents the averaged thickness of 5 random fields from 1 mouse. Data are presented as mean ± SD and analyzed using 2-way ANOVA followed by Tukey’s multiple comparisons test. TY (*n* = 4 mice), TP (*n* = 3 mice), TCD (*n* = 5 mice), TPCD (*n* = 4 mice). (**F**) Representative H&E staining of stomach tissues after tamoxifen administration. Scale bar, 200 μm. (**G**) Pathological staging of stomach cancer progression based on infiltration of tumor cells in TPCD mice. (**H**) Representative H&E staining of small and large intestines in TPCD mice. Scale bar, 1 mm.

To investigate the functional consequences of *Kmt2c*, *Kmt2d*, and *Pten* loss, we utilized *Tmprss2-CreER^T2^-IRES-nlsEGFP* (referred to as *Tmprss2-CreER^T2^* hereafter) to mediate tamoxifen-induced deletion of floxed *Kmt2c*, *Kmt2d*, and *Pten* alleles in epithelial cells of prostate, bladder, and gastrointestinal tract (10, 25, 26). Consistent with other stomach epithelium–specific Cre (27), *Tmprss2-CreER^T2^* induced *LoxP* recombination in the majority of epithelial cells but not in stromal cells in *Rosa26-CAG-LSL-EYFP* (TY) mice (Figure 2, A and B).

We then crossed *Tmprss2-CreER^T2^* with *Kmt2c^fl/fl^* (TC), *Kmt2d^fl/fl^* (TD), the combination (TCD), and all with *Pten^fl/fl^* to generate TP, TPC, TPD, and TPCD mouse models, respectively. The deletion efficiency of the conditional *Kmt2c* and *Kmt2d* alleles in each model was validated by in situ hybridization (BaseScope) of the floxed exons and by immunohistochemistry (IHC) against H3K4me1 (Supplemental Figure 2A). To assess the deletion efficiency of the conditional *Pten* allele, we performed IHC against PTEN and serine 473 and threonine 308 phosphorylated AKT (p-AKT) and observed widespread epithelial PTEN loss and AKT phosphorylation in the stomachs of TP and TPCD mice (Supplemental Figure 2A).

Our prior work showed that TC, TD, and TCD mice had normal lifespans, whereas TPC and TPD male mice lived to ~6 and ~9 months, respectively, due to urothelial cancer leading to urinary obstruction and renal failure. In contrast, TPCD mice survived for only 6 weeks after tamoxifen administration due to stomach cancer and malnutrition (10). Here, we confirmed the posttamoxifen survival rates of TY, TP, TCD, and TPCD mice in an independent cohort (Figure 2C) and collected stomachs for histology analysis at several time points in concordance with their survival (Supplemental Table 1).

We examined the stomach tissues at the 12-month time point for TC and TD groups and at the 6-month time point for TPC and TPD groups (Supplemental Figure 3A). Only minor histological changes were observed in the gastric mucosa of TC and TD groups at 12 months (Supplemental Figure 3B), whereas both TPC and TPD groups exhibited dysplasia at 6 months. In TPD mice, there were focal lesions that progressed to carcinoma in situ (CIS) (Supplemental Figure 3, C and D). IHC further showed that regions of dysplasia and CIS exhibited loss of PTEN and H3K4me1 staining and gain of p-AKT staining, while histologically normal regions maintained PTEN and H3K4me1 staining (Supplemental Figure 3E). These data indicate that loss of *Kmt2c* or *Kmt2d* alone is insufficient to cause observable histologic dysplasia but cooperates with *Pten* loss in gastric tumorigenesis; they further suggest that *Kmt2d* loss may be more potent than *Kmt2c* loss in promoting gastric cancer initiation.

To examine early direct effects, we focused our analyses on TP, TCD, and TPCD mice at the 3-week and 6-week time points. In TP, TCD, and TPCD groups, we observed progressively increased gross weight of whole stomach and increased thickness of stomach mucosa (Figure 2, D–F, and Supplemental Figure 4A). Histological analysis revealed mucosal hyperplasia in TP, nuclear dysplasia in TCD, and muscle-invasive gastric cancer in all TPCD stomachs. The cancer exhibits histologic features of Lauren intestinal type (5). We observed invasion into the submucosa (pT1), the muscularis (pT2), and the serosa (pT3) (Figure 2G and Supplemental Figure 5, A–C). The cancers were highly inflammatory with infiltration of lymphocytes, formation of secondary lymphoid structures, and enlargement of draining lymph nodes (Supplemental Figure 4A and Supplemental Figure 5C). We performed E-cadherin staining that highlighted the infiltrating tumor cells into the submucosa but did not show any lymph node metastasis at this early time point (Supplemental Figure 6, A–C). Although *Tmprss2-CreER^T2^* also induces *LoxP* recombination in epithelial cells of small and large intestines (25), we did not observe any tumors in these tissues at the 6-week time point despite loss of PTEN and H3K4me1 on IHC (Figure 2H and Supplemental Figure 7A). This is consistent with the low rate of *KMT2C/D* LOF mutations in colorectal adenocarcinoma (Figure 1B), indicating that *KMT2C/D* are likely selective tumor suppressors in STAD.

### Kmt2c/d knockout impairs gastric lineage differentiation and promotes intestinal metaplasia.

**Figure 3:**
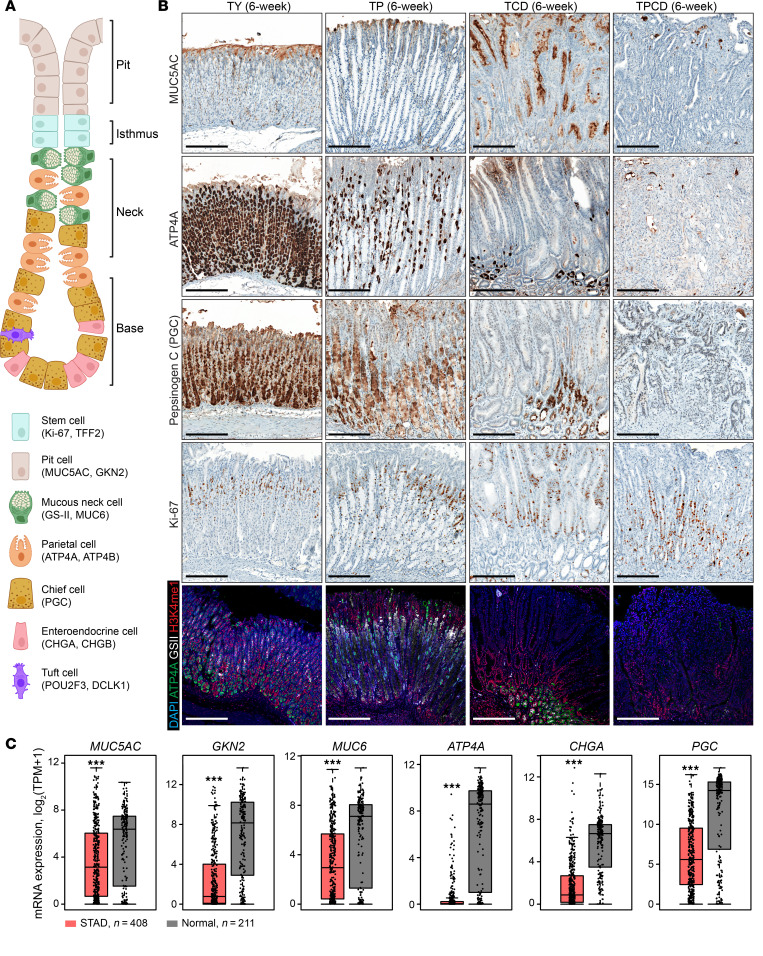
*Kmt2c/d* knockout impairs gastric differentiation. (**A**) Schematic diagram illustrating cell types and their distribution in mouse stomach corpus (created with BioRender.com). (**B**) Top, representative IHC staining of MUC5AC, ATP4A, PGC, and Ki-67 in stomach tissues. Bottom, representative IF of ATP4A, H3K4me1, and lectin GS-II in stomach tissues. Scale bar, 200 μm. (**C**) Expression of *MUC5AC*, *GKN2*, *MUC6*, *ATP4A*, *CHGA*, and *PGC* in STAD (shown in red) and normal stomach (gray) from TCGA dataset. Data were extracted using GEPIA2. The center line represents the median, the box limits represent the upper and lower quartiles, and the whiskers represent 1.5 × the interquartile range. Statistical significance was determined using 2-tailed *t* test, ***P < 0.001.

The stomach is composed of gastric glands consisting of pit cells on the surface that form a barrier, stem cells that proliferate and give rise to the other cell types, mucous neck cells that secrete mucus, parietal cells that secrete hydrochloric acid, chief cells that secrete digestive enzymes, enteroendocrine cells that secrete gut hormones, and tuft cells that sense the local environment (Figure 3A) (28). To examine cellular differentiation in GEMMs, we performed IHC and immunofluorescence (IF) staining of gastric lineage markers, including oligomeric mucus/gel-forming (MUC5AC) (pit cells), ATPase H+/K+ transporting subunit alpha (ATP4A) (parietal cells), pepsinogen C (PGC, chief cells and mucous neck cells), and lectin Griffonia simplicifolia-II (GSII, mucous neck cells). In TP mice, we observed mucous neck cell hyperplasia (29), characterized by increased GSII-positive mucous neck cells and decreased ATP4A-positive parietal cells (Figure 3B). Ki-67–positive stem/progenitor cells remained in the isthmus region above the mucous neck cells and appeared slightly expanded (Figure 3B). In TCD mice, we observed nuclear dysplasia in the apical region, marked by increased number and proliferation of MUC5AC-positive pit cells (Figure 3B) (29). In TPCD mice, we observed the loss of most gastric lineage makers, along with a disorganized pattern of Ki-67–positive proliferating cells (Figure 3B). Compared with normal tissues, the expression of gastric lineage markers was also significantly reduced in human STAD samples (Figure 3C).

**Figure 4:**
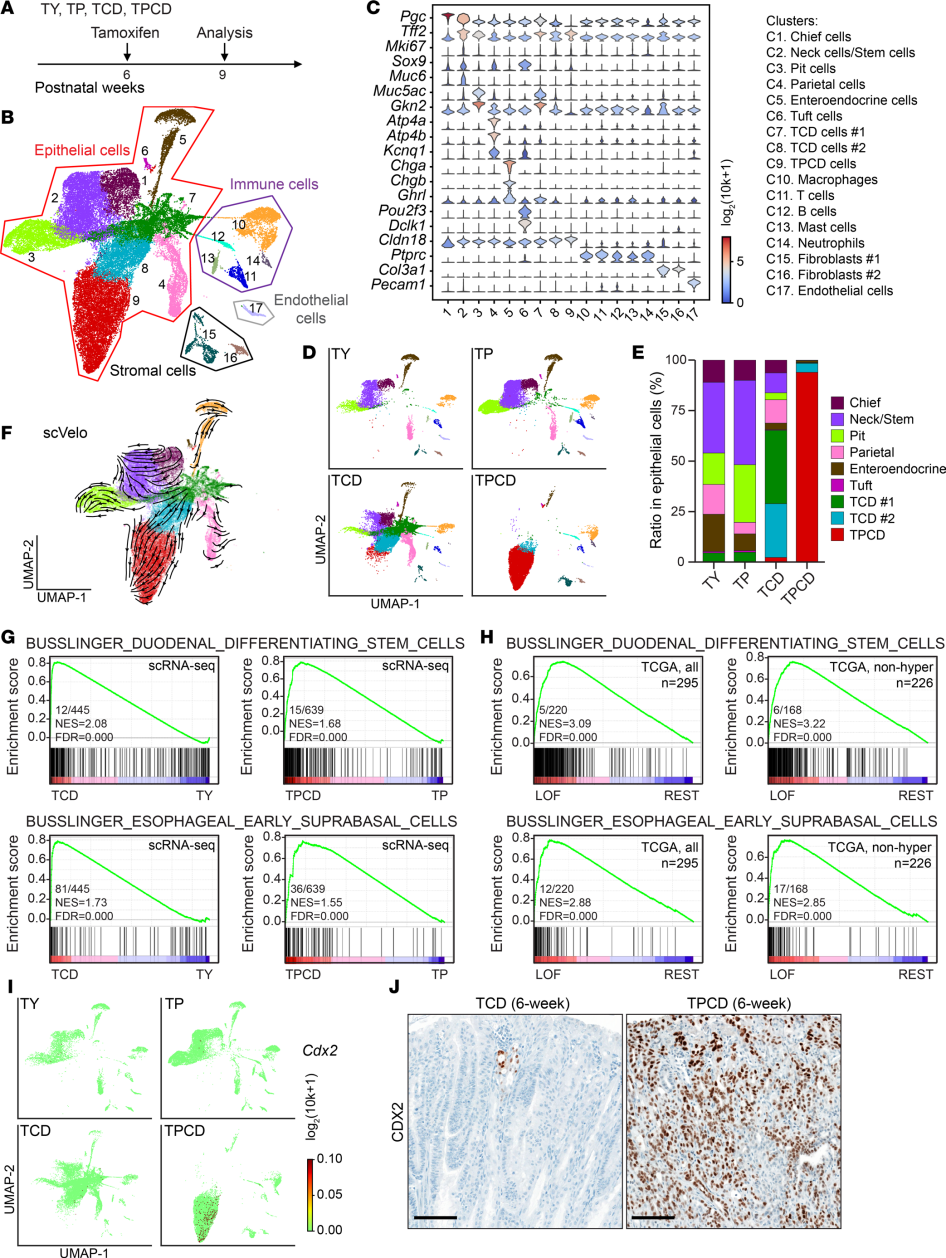
scRNA-seq reveals impaired differentiation after *Kmt2c/d* loss. (**A**) Schematic of mouse models: 2 doses of tamoxifen (3 mg × 2) were injected intraperitoneally with a 48-hour interval. Tissues were collected 3 weeks after tamoxifen administration. (**B**) Uniform manifold approximation projection (UMAP) plots showing cell clusters in pooled TY, TP, TCD, and TPCD mice. (**C**) Violin plots of representative cell cluster marker genes. The color in the violin plots indicates the median normalized expression level of genes. (**D**) UMAP plots showing cell clusters in TY, TP, TCD, and TPCD mice. (**E**) Percentage of each cell cluster in the pooled epithelial components (C1–C9). (**F**) UMAP-based embedding of RNA velocity analysis showing trajectory transition among cell clusters. (**G**) GSEAs in scRNA-seq showing positive enrichment of gene sets associated with duodenal and esophageal lineages following *Kmt2c/d* deletion. (**H**) GSEAs in human TCGA STAD dataset showing positive enrichment of gene sets associated with duodenal and esophageal lineages in *KMT2C/D*-LOF samples. (**I**) UMAP color-coded by expression of *Cdx2* in TY, TP, TCD, and TPCD cell clusters. (**J**) Representative IHC of CDX2 in stomach tissues of TCD and TPCD mice. Scale bar, 200 μm.

To characterize the diversity of transcriptional alterations, we performed single-cell RNA sequencing (scRNA-seq) on dissociated stomach mucosa 3 weeks after tamoxifen administration (Figure 4A). Viable cells were sorted as DAPI-negative using FACS (Supplemental Figure 8A). Cell types were identified using marker genes from prior works and PanglaoDB (28, 30, 31). We analyzed 47,777 single-cell transcriptomes from TY (*n* = 2), TP (*n* = 3), TCD (*n* = 3), and TPCD (*n* = 2) mice (Figure 4B and Supplemental Figure 8B), identifying cell clusters using uniform manifold approximation projection (UMAP) and Leiden clustering.

In normal stomachs, we identified clusters of chief cells marked by high *Pgc* expression (cluster C1); neck cells/stem cells marked by high*Tff2*, *Muc6*, and *Mki67* expression (C2); and pit cells marked by high *Muc5ac* and *Gkn2* expression (C3) that form a continuum (Figure 4, B–D, and Supplemental Figure 8, C and D). Neck cells and stem cells were placed into the same cluster, but examination of neck cell marker (*Muc6*) and stem cell markers (*Mki67*, *Tff2*) showed that they occupied different regions of the cluster on UMAP (Supplemental Figure 8D). We further identified parietal cells marked by *Atp4a*, *Atp4b*, and *Kcnq1* (C4); enteroendocrine cells marked by *Chga*, *Chgb*, and *Ghrl* (C5); and tuft cells marked by *Pou2f3* and *Dclk1* (C6) (Figure 4, B–D, and Supplemental Figure 8, C and D).

In the presence of *Pten* knockout, we observed no new cell clusters but increased ratio of both pit cell and neck/stem cell clusters, consistent with the phenotype of mucous neck cell hyperplasia (Figure 3B and Figure 4E). After *Kmt2c/d* knockout, we identified new cell clusters C7 and C8, as well as C9 in the presence of *Pten* deletion. Cluster C7 exhibited lineage infidelity, with mixed expression of pit cell markers *Muc5ac* and *Gkn2*, neck cell marker *Tff2*, enteroendocrine cell marker *Ghrl*, and chief cell marker *Pgc* (Figure 4C and Supplemental Figure 8D), aligning with the phenotype of nuclear dysplasia (Figure 3B). Both clusters C7 and C9 exhibited high expression of *Tff2* (Figure 4C), a marker of isthmus progenitor cells (32). In TPCD mice, the dominant cluster C9 showed loss of most gastric epithelial lineage markers (Figure 4C and Supplemental Figure 8D), consistent with the dedifferentiation in histological observations. There was upregulation of many genes that are known to be upregulated in human stomach cancer, including claudin-18 (*Cldn18*), *Onecut2*, *Cldn4*, *Trop2*, *Klf5*, *Cftr*, and *Plaur*. Interestingly, *Cldn18* was upregulated in clusters C8 and C9 (Figure 4C and Supplemental Figure 8D), suggesting the potential sensitivity of *KMT2C/D*-deficient tumors to CLDN18-based therapies (33). UMAP-based embedding of RNA velocity analysis revealed the transitional trajectory linking the new clusters C7, C8, and C9 to normal stomach epithelial lineages (Figure 4F).

To explore the transcriptional alterations in differentiation, we pooled single-cell transcriptomes of all gastric epithelial cells of each genotype and performed gene set enrichment analysis (GSEA) using cell type signature gene sets (C8) from single-cell sequencing studies of human tissues. We focused on the transcriptional perturbations induced by *Kmt2c/d* knockout. Gene sets associated with duodenal and esophageal differentiation were positively enriched in both TP versus TY and TPCD versus TP comparisons (Figure 4G), suggesting the induction of dedifferentiation or transdifferentiation following *Kmt2c/d* loss. GSEA of TCGA STAD data also showed positive enrichment of duodenal and esophageal lineage markers in samples with LOF mutations in *KMT2C/D* when we included the entire dataset or when we excluded MSI samples (nonhyper), suggesting this is not an MSI-specific phenomenon (Figure 4H and Supplemental Figure 9, A and B). We performed Alcian blue staining of acidic mucin. Alcian blue stains goblet cells in normal stomach and is clinically used to identify intestinal metaplasia (29). We detected Alcian blue–positive cells in deep antral gland but not in corpus gland of TY mice (Supplemental Figure 9C), consistent with prior reports (29, 34). In contrast, aberrant Alcian blue–positive staining in suprabasal stomach tissues was observed in TP, TCD, and TPCD mice, reflecting the altered expression of mucins. Clinically, the intestinal lineage transcriptional factor CDX2 is found in premalignant gastric lesions that exhibit intestinal metaplasia (IM) and in intestinal subtype gastric carcinoma (35). We observed the expression of CDX2 in TCD and TPCD mice, suggesting the intestinal differentiation in our models (Figure 4, I and J). Together, these data highlight the hyperplastic and metaplastic lineage changes following *Pten* or *Kmt2c/d* loss.

**Figure 5:**
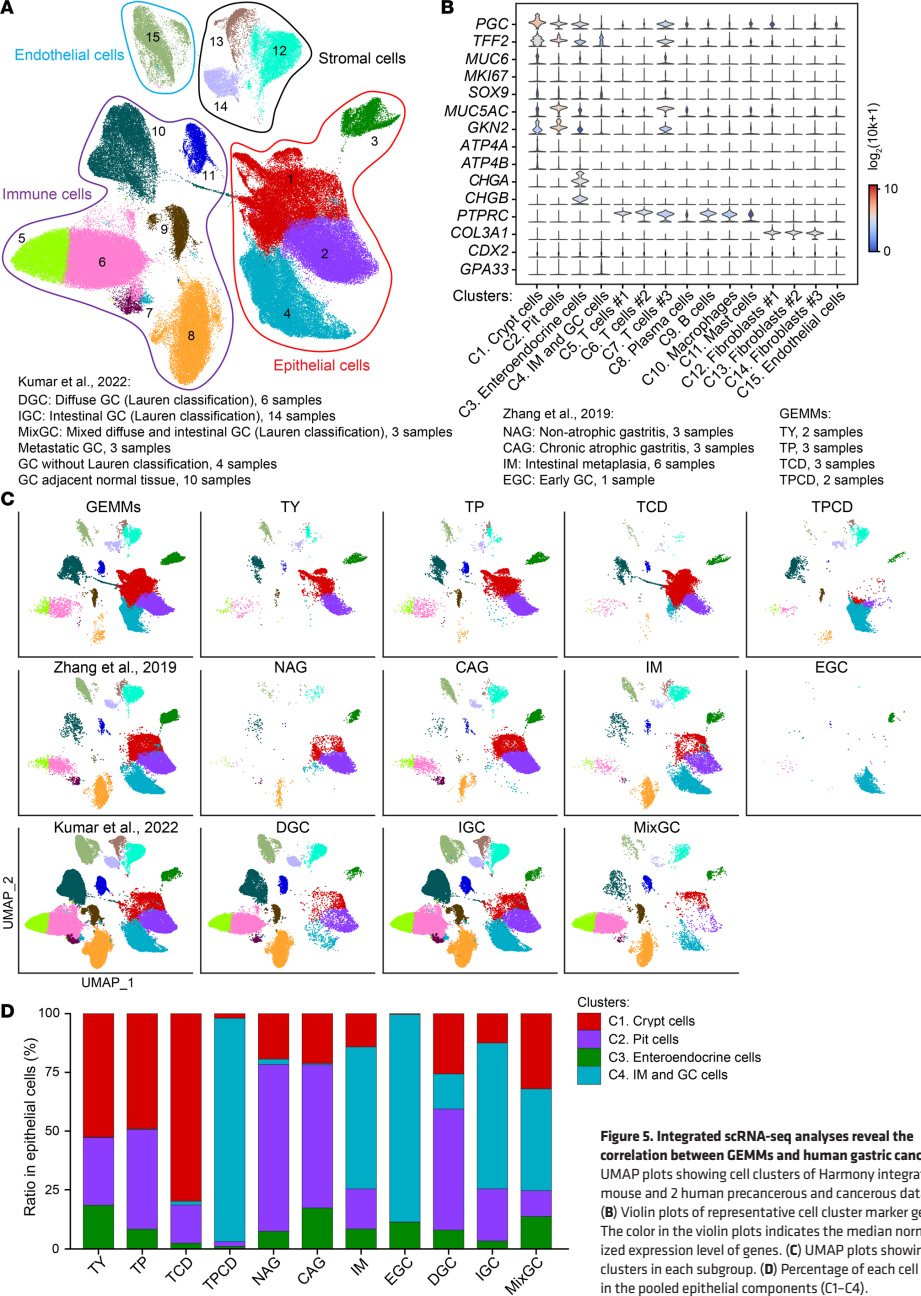
Integrated scRNA-seq analyses reveal the correlation between GEMMs and human gastric cancer. (**A**) UMAP plots showing cell clusters of Harmony integrated mouse and 2 human precancerous and cancerous data sets. (**B**) Violin plots of representative cell cluster marker genes. The color in the violin plots indicates the median normalized expression level of genes. (**C**) UMAP plots showing cell clusters in each subgroup. (**D**) Percentage of each cell cluster in the pooled epithelial components (C1–C4).

To further investigate the correlation between GEMMs and human gastric cancer development, we conducted integrated analyses of scRNA-seq data with human precancerous and cancerous stomach samples from 2 recently published datasets (36, 37), using Harmony (38). Based on the UMAP visualization of the integrated data, we identified 4 major groups of cell clusters: stromal cells, immune cells, endothelial cells, and epithelial cells (Figure 5, A and B). There was strong overlap among all cell populations within the endothelial, stromal, and immune clusters, reinforcing confidence in the analysis. Here, we focused on the epithelial compartment and included samples of TY, TP, TCD, TPCD, nonatrophic gastritis (NAG), chronic atrophic gastritis (CAG), IM, early gastric cancer (EGC), diffuse gastric cancer (DGC), intestinal gastric cancer (IGC), and mixed diffuse and intestinal gastric cancer (MixGC) (Figure 5, A–C). In TCD mice, there was expansion of cluster C1 comprising crypt cell types and extension of few cells into cluster C4 that consists of human gastric IM and intestinal carcinoma cells (Figure 5, C and D). In TPCD mice, the vast majority of epithelial cells clustered into cluster C4 (Figure 5, C and D). These data suggest that the gastric epithelium of TPCD faithfully recapitulates the transcriptional program of intestinal-type gastric cancer.

### Kmt2c/d loss enhances MHC class I expression and antigen presentation.

**Figure 6:**
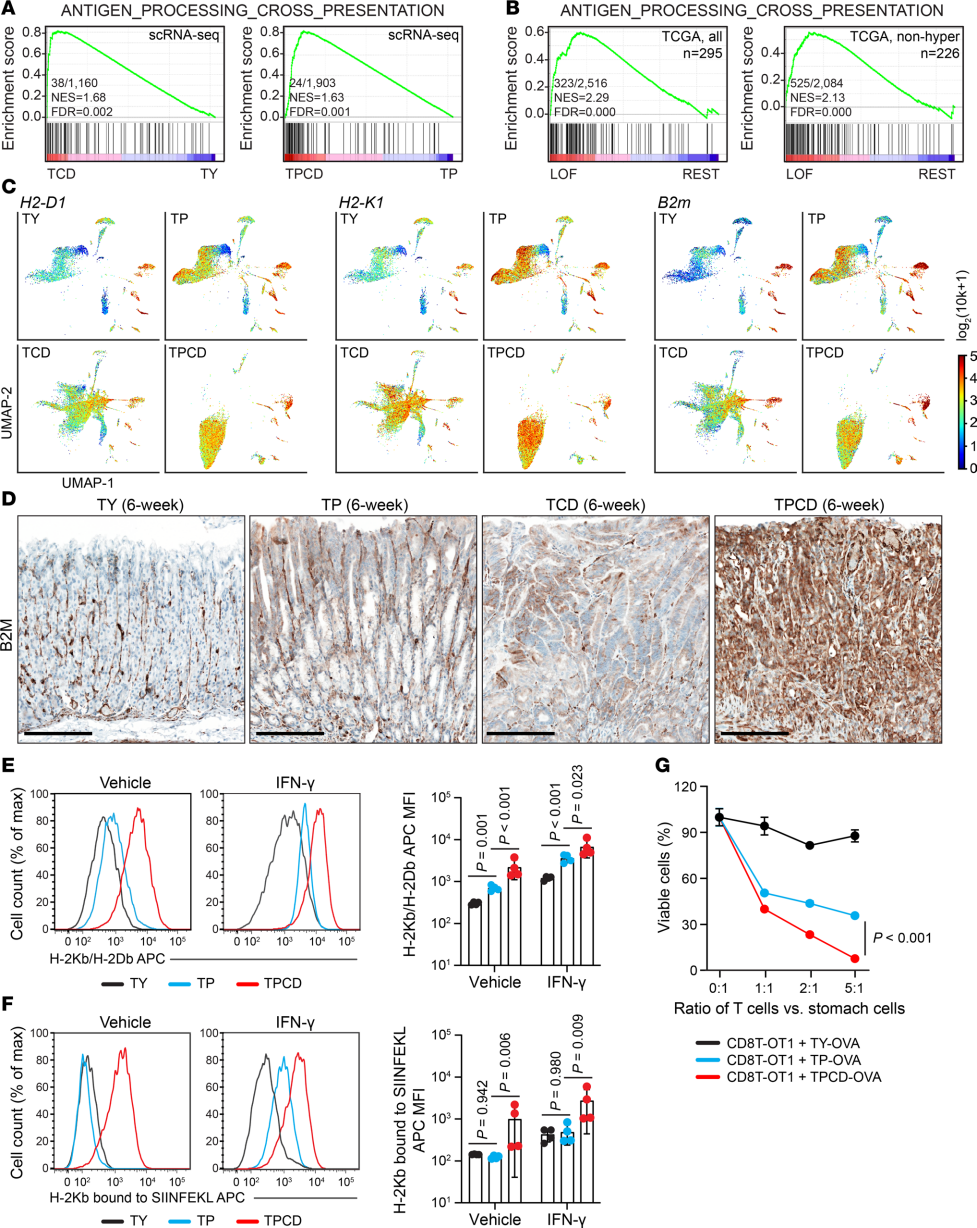
*Kmt2c/d* loss enhances MHC-I expression and antigen presentation. (**A**) GSEAs in scRNA-seq showing positive enrichment of gene set associated with antigen presentation following *Kmt2c/d* deletion. (**B**) GSEAs in human TCGA STAD showing positive enrichment of gene sets associated with antigen presentation in *KMT2C/D*-LOF samples. (**C**) UMAP color-coded by expression of *H2-D1*, *H2-K1*, and *B2m* in TY, TP, TCD, and TPCD cell clusters. (**D**) Representative IHC of B2M in stomachs of TY, TP, TCD, and TPCD mice. Scale bar, 200 μm. (**E**) Flow cytometry analysis of MHC class I molecules H-2Kb/H2-Db in cultured stomach epithelial cells. To induce the expression of H-2Kb/Db, cells were treated with vehicle or mouse interferon-gamma (IFN-γ, 10 ng/mL) for 24 hours. Median fluorescent intensity values are shown on a logarithmic scale due to the wide dynamic range and right-skewed distribution of flow cytometry data, enabling appropriate visualization of fold-changes. Data are presented as mean ± SD (*n* = 4) and analyzed using 2-way ANOVA followed by Tukey’s multiple comparisons test on log_10_-normalized data. (**F**) Flow cytometry analysis of H-2Kb bound SIINFEKL in OVA-expressing stomach epithelial cells. To enhance antigen presentation, cells were treated with vehicle or mouse IFN-γ (10 ng/mL) for 24 hours. Median fluorescent intensity values are shown on a logarithmic scale due to the wide dynamic range and right-skewed distribution of flow cytometry data, enabling appropriate visualization of fold-changes. Data are presented as mean ± SD (*n* = 4) and analyzed using 2-way ANOVA followed by Tukey’s multiple comparisons test on log_10_-normalized data. (**G**) Viable OVA-expressing stomach cells after coculture with OT1 CD8^+^ T cells. Data are presented as mean ± SD (*n* = 3) and analyzed using 2-way ANOVA followed by Tukey’s multiple comparisons test in the 5:1 group (T cells/stomach cells = 5:1).

Next, we sought to explore potential therapeutic opportunities arising from *Kmt2c/d* loss. GSEA of scRNA-seq data revealed that a gene set associated with antigen presentation was positively enriched after *Kmt2c/d* knockout in both wild-type and *Pten*-loss background (Figure 6A). Consistently, comparison of gastric cancers with *KMT2C/D* LOF mutations versus other gastric cancers in TCGA STAD samples also showed positive enrichment of this gene set, even when MSI samples were removed from the analysis (Figure 6B and Supplemental Figure 10, A and B). Compared with TY, upregulated expression of MHC class I (MHC-I) components *H2-D1*, *H2-K1*, and *B2m* was identified in TP, TCD, and TPCD groups (Figure 6C). The upregulated B2M protein level was confirmed by IHC staining (Figure 6D). Notably, in TPCD mice with heterogenous *Kmt2c/d* deletion, higher B2M staining intensity was observed in *Kmt2c/d*-deficient neoplastic epithelial cells compared with the adjacent *Kmt2c/d*-intact histologically normal cells (Supplemental Figure 10C), suggesting that the increased B2M level was primarily caused by *Kmt2c/d* deletion rather than the microenvironment. The upregulated MHC-I was associated with increased secondary lymphoid structures (e.g., Peyer’s patches) in TCD and TPCD stomachs (Supplemental Figure 4A and Supplemental Figure 5C).

In the TCGA STAD dataset, *B2M* expression was markedly upregulated in samples lacking *KMT2D* or lacking both *KMT2C* and *KMT2D* (Supplemental Figure 10D). To investigate the distinct effects of *Kmt2c* or *Kmt2d* single loss on MHC-I expression, we used CRISPR/Cas9 to knock out *Kmt2c* and/or *Kmt2d* in TP cells (Supplemental Figure 10E). Consistent with prior report (10), *Kmt2d* knockout and *Kmt2c/d* double knockout decreased H3K4me1 modification but did not affect the levels of H3K4me3 or KMT2A/B proteins (Supplemental Figure 10F). Flow cytometry analysis showed that *Kmt2c* or *Kmt2d* loss caused significant upregulation of H2-Kb and H2-Db expression (Supplemental Figure 10G). We further identified that the loss of *Kmt2c* or *Kmt2d* does not affect the expression of the other gene (Supplemental Figure 10, H and I), suggesting no transcriptional compensation between these 2 genes.

To assess antigen presentation capability, we exogenously expressed chicken ovalbumin (OVA) in TY, TP, and TPCD stomach organoids. Gastric epithelial cells in the TCD group grew poorly in vitro and could not be studied (Supplemental Figure 11A). We performed PCR using DNA isolated from organoids to confirm efficient deletion of the appropriate alleles (Supplemental Figure 11, B and C). We used flow cytometry to quantify the expression of MHC-I molecules and the presentation of antigen SIINFEKL in TY, TP, and TPCD cells at baseline and after interferon-gamma (IFN-γ) stimulation. Consistent with scRNA-seq data, we observed progressively increased cell surface expression of H-2Db and H-2Kb at baseline and after IFN-γ stimulation in TP and TPCD cells (Figure 6E). Presentation of SIINFEKL, the OVA-derived peptide (OVA257-264), by H-2Kb MHC-I molecule, was significantly enhanced with *Kmt2c/d* knockout (Figure 6F). To functionally evaluate the susceptibility to antigen-specific T cell–mediated cytotoxicity, we assayed the coculture of OVA-expressing stomach cells with OT1 CD8^+^ T cells that recognize the SIINFEKL peptide presented by H-2Kb MHC-I. We observed significantly increased T cell–mediated cytotoxicity of TPCD compared with TP cells (Figure 6G). These data suggest that *Kmt2c/d* knockout context in gastric cancer is primed for augmented antigen presentation, implying potential sensitivity to immunotherapy, consistent with a prior study in liver cancer (39).

### Kmt2c/d loss reduces new protein synthesis.

**Figure 7:**
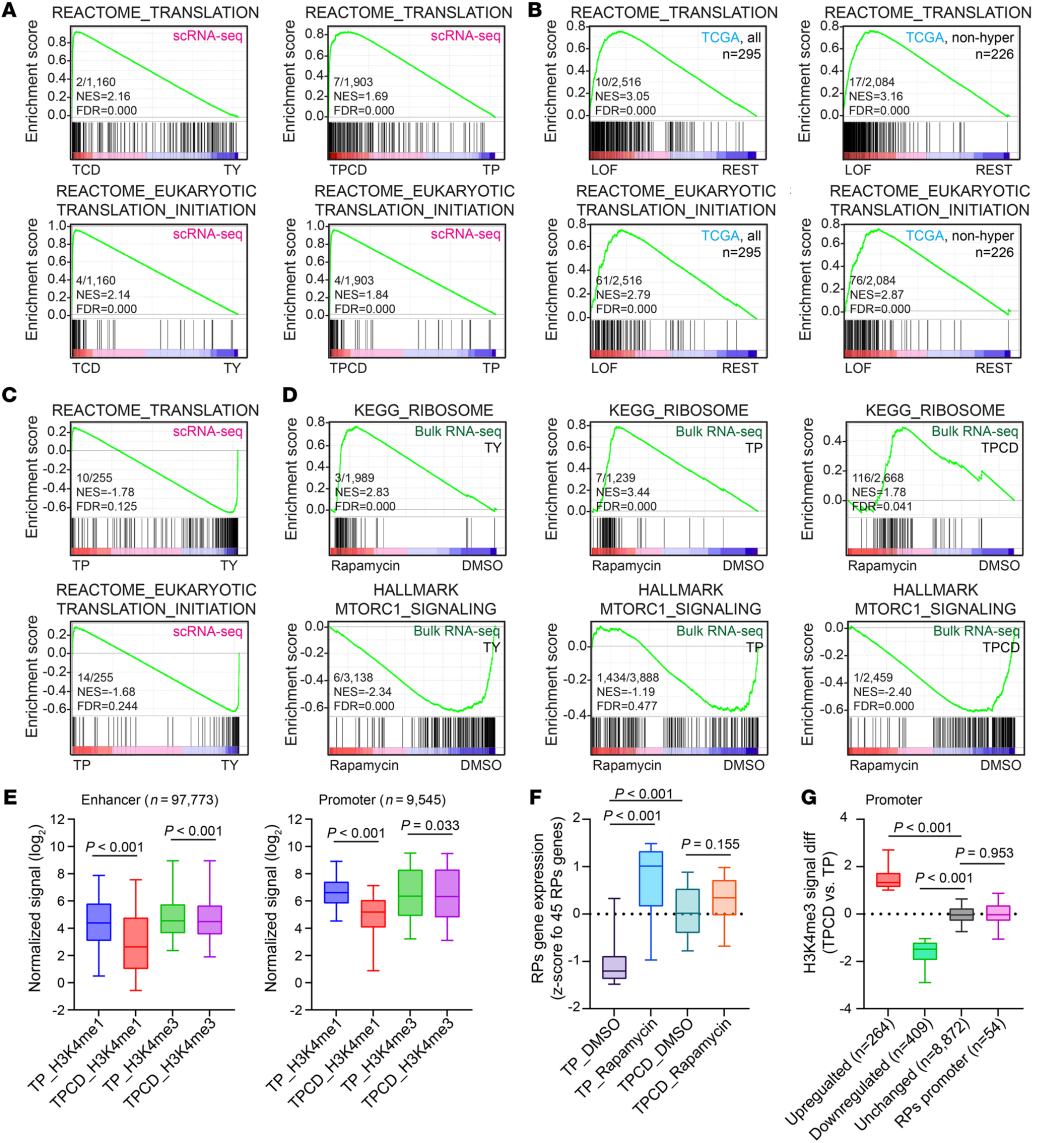
*Kmt2c/d* loss induces feedback upregulation of ribosomal protein expression due to inadequate translation. (**A**) GSEAs in scRNA-seq showing positive enrichment of gene sets associated with protein translation following *Kmt2c/d* deletion. (**B**) GSEAs in human TCGA STAD showing positive enrichment of gene sets associated with protein translation in *KMT2C/D*-LOF samples. (**C**) GSEAs in scRNA-seq showing negative enrichment of gene sets associated with protein translation following *Pten* deletion. (**D**) GSEAs in bulk RNA-seq of TY, TP, and TPCD stomach epithelial cells. Rapamycin suppressed the expression of mTORC1 target genes but upregulated expression of RPs. (**E**) H3K4me1 and H3K4me3 peaks and intensity at enhancers and promoters in TP and TPCD cells. The center line represents the median, the box limits represent the upper and lower quartiles, and the minimum and maximum whiskers represent the 5th and 95th percentiles, respectively. Statistical significance was determined using unpaired 2-tailed *t* test with Welch’s correction. (**F**) *Z*-score of 45 selected RP gene expression in bulk RNA-seq of TP and TPCD cells upon rapamycin treatment (10 nM, 24 hours). RP genes with identified promoter H3K4me3 peaks were included in the analysis. The center line represents the median, and the box limits represent the 5th and 95th percentiles, respectively. Statistical significance was determined using unpaired 2-tailed *t* test with Welch’s correction. (**G**) H3K4me3 signal change (log_2_ diff) between TP and TPCD cells. Promoter H3K4me3 peaks were categorized as upregulated (*n* = 264), downregulated (*n* = 409), unchanged (*n* = 8,872), and pooled RP genes (*n* = 54). The center line represents the median, the box limits represent the upper and lower quartiles, and the minimum and maximum whiskers represent the 5th and 95th percentiles, respectively. Statistical analysis was performed using 1-way ANOVA followed by Dunnett’s multiple comparisons test.

In addition to augmented antigen presentation, we observed that gene sets REACTOME_TRANSLATION and REACTOME_EUKARYOTIC_TRANSLATION_INITIATION were among the most positively enriched gene sets when comparing TCD versus TY and TPCD versus TP in mouse scRNA-seq data (Figure 7A). These gene sets primarily consist of ribosomal proteins (RPs), which are involved in ribosomal biogenesis and translation. In the TCGA STAD dataset, they were enriched in samples with LOF mutations of *KMT2C* and/or *KMT2D* (Figure 7B and Supplemental Figure 12, A and B). Interestingly, in *Pten*-loss cells, known to activate mTOR signaling and enhance translation, we found negative enrichment of gene sets associated with protein synthesis (Figure 7C). To directly evaluate new protein synthesis, we performed puromycin incorporation assay in the stomachs of TCD mice in vivo. Compared with H3K4me1-high cells, reduced puromycin staining intensity was observed in H3K4me1-low cells (Supplemental Figure 12C), indicating decreased new protein synthesis after *Kmt2c/d* loss. We further used O-propargyl-puromycin (OPP), an analog of puromycin, to compare protein synthesis in vitro via flow cytometry. Consistent with a prior report (40), *Pten* deletion significantly increased new protein synthesis (Supplemental Figure 12, D and E), while *Kmt2c/d* knockout reduced protein synthesis in TPCD cells (Supplemental Figure 12, D and E). In *Pten*-loss context, *Kmt2d* knockout caused a more substantial reduction in nascent protein synthesis than *Kmt2c* loss (Supplemental Figure 12F), corroborating with the greater decrease of H3K4me1 upon *Kmt2d* knockout (Supplemental Figure 10F).

To determine the mechanism by which *Kmt2c/d* loss regulates RPs’ expression, we treated TY, TP, and TPCD stomach organoids with the mTORC1-selective inhibitor rapamycin and performed bulk RNA-seq. While rapamycin effectively suppressed the expression of mTORC1 downstream genes in TY, TP, and TPCD cells (Figure 7D), it paradoxically triggered the upregulation of RPs’ genes, as evidenced by the positive enrichment of the KEGG_RIBOSOME gene set (Figure 7D). These data suggest a compensatory mechanism between protein synthesis and RP expression.

We further performed chromatin immunoprecipitation with sequencing (ChIP-seq) of H3K4me1 and H3K4me3 in TP and TPCD cells. *Kmt2c/d* knockout induced a decrease of H3K4me1 modification at enhancers (*n* = 97,773) and promoters (*n* = 9,545) (Figure 7E and Supplemental Figure 13, A and B), with only a minor reduction of H3K4me3 at these regions (Figure 7E and Supplemental Figure 13, A and B). A pooled analysis of 45 RPs’ genes showed higher baseline expression in TPCD cells and a further increase upon rapamycin treatment in both genotypes (Figure 7F). Notably, H3K4me3 signal at RP gene promoters showed no significant difference between TP and TPCD cells (Figure 7G), indicating that the upregulation of these genes is not a direct consequence of altered histone methylation at their promoters.

Together, these data indicate that in the gastric epithelium, there is an inverse correlation between the mRNA expression of RP genes and the overall rate of protein translation and posit that upregulation of gene sets associated with translation upon *Kmt2c/d* loss, mostly comprising RPs, may be a compensatory response to inadequate translation (41).

### Kmt2c/d loss confers sensitivity to mTORC1 inhibition.

**Figure 8:**
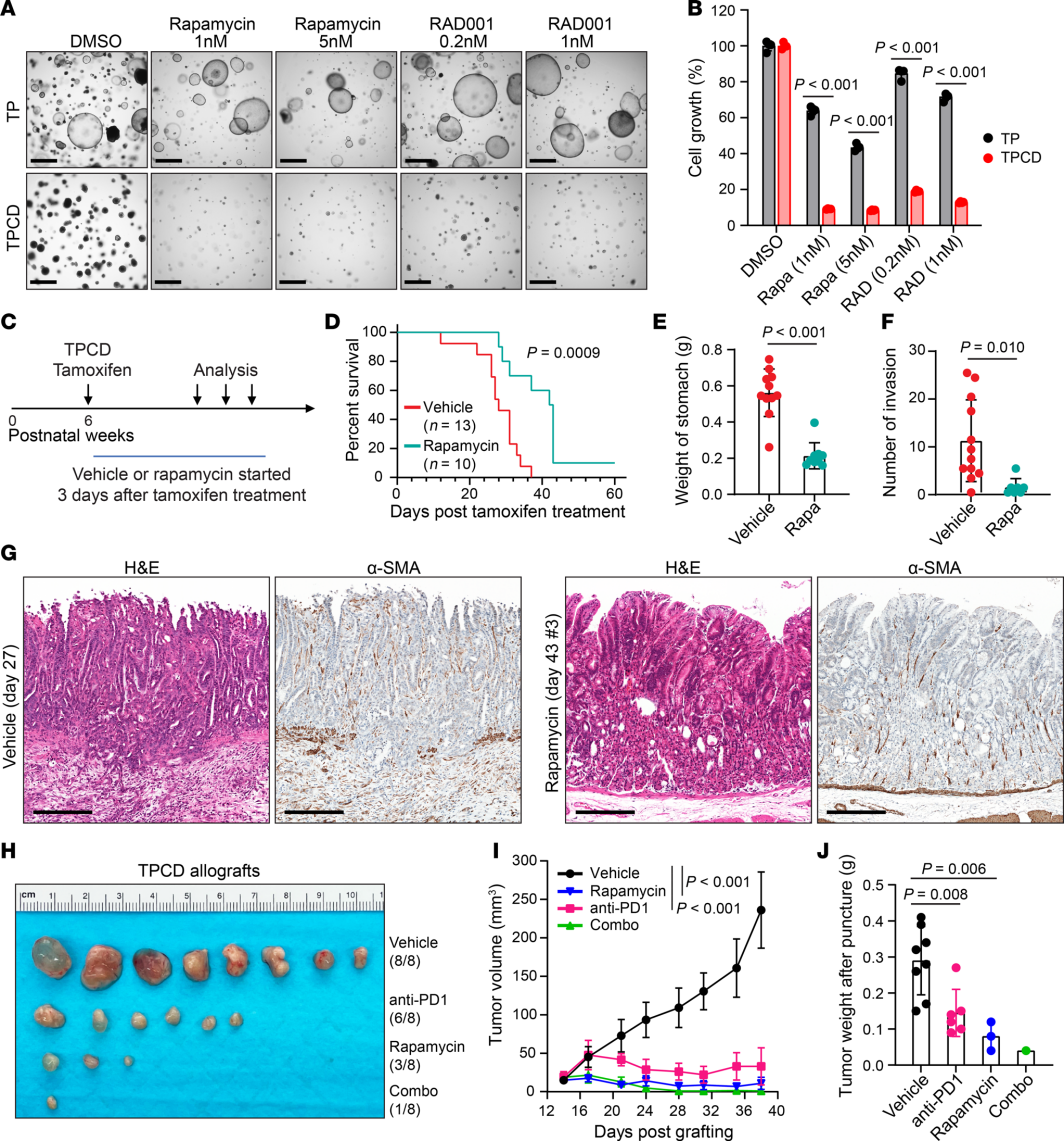
Combination of rapamycin and anti–PD-1 suppresses growth of TPCD cells in vivo. (**A** and **B**) Representative bright-field images of organoid from TP and TPCD groups. Scale bar, 1 mm. Cells were seeded in Matrigel (500 cells per blob, 50 μL), then treated from day 2 for 10 days. Organoid culture medium and inhibitors were refreshed every 4 days. Cell growth was measured using the CellTiter-Glo luminescent reagent. Data are presented as mean ± SD (*n* = 3) and analyzed with 2-tailed *t* test. (**C**) Schematic illustration showing the induction and treatment of stomach cancer in TPCD mice. Vehicle or rapamycin treatment started 3 days after the first dose of tamoxifen. (**D**) Kaplan-Meier plots showing the survival of mice treated with vehicle or rapamycin. (**E**) Stomach weight in TPCD mice treated with vehicle (*n* = 13 mice) or rapamycin (*n* = 10 mice). Data are presented as mean ± SD and analyzed with 2-tailed *t* test. (**F**) Statistics of invasive lesions in TPCD mice treated with vehicle (*n* = 13 mice) or rapamycin (*n* = 10 mice). Invasion sites were determined using IHC of α–smooth muscle actin (α-SMA). Data are presented as mean ± SD and analyzed with 2-tailed *t* test. (**G**) Representative H&E and IHC of α-SMA in vehicle- or rapamycin-treated TPCD mice. Scale bar, 200 μm.#3, third mouse. (**H** and **I**) Allografts and growth curves of TPCD tumors treated with rapamycin (5 mg/kg/d) or anti–PD-1 (8 mg/kg, twice a week) in C57BL/6 mice (*n* = 8 grafts per condition). Treatment started 2 weeks after injection of cells into the mammary fat pad. Data are presented as mean ± SEM and analyzed with 2-tailed *t* test at endpoint. (**J**) Statistics of TPCD tumor weight in C57BL/6 mice. Data are presented as mean ± SD and analyzed with 2-tailed *t* test.

Our data indicate that *Kmt2c/d* loss leads to compromised protein translation; we subsequently tested whether this may lead to sensitivity to PI3K/AKT/mTOR pathway inhibitors. We performed cell viability assay using PI3Kα inhibitor (BYL-719), PI3Kβ inhibitor (AZD8186), AKT inhibitor (ipatasertib), mTORC1-specific inhibitors (rapamycin and RAD001), and dual mTORC1/2 inhibitor (INK-128). TPCD cells were more sensitive to mTOR inhibitors than TP cells, with greater difference to mTORC1-specific inhibitors (Figure 8, A and B, and Supplemental Figure 14A). In contrast, TP and TPCD cells showed no differential responses to PI3K and AKT inhibitors (Supplemental Figure 14A). Western blot analyses confirmed that mTORC1 inhibitors were more potent and specific at inhibiting downstream 4EBP1 and p70 S6 kinase phosphorylation (Supplemental Figure 14B). These data suggest that the therapeutic vulnerability conferred by *Kmt2c/d* loss is specific to mTORC1 inhibition and is not specifically affected by the upstream PI3K/AKT pathway.

To determine if rapamycin can affect gastric cancer development in TPCD GEMM, we treated TPCD mice with vehicle or rapamycin starting 3 days after tamoxifen administration (Figure 8C). Rapamycin treatment significantly prolonged the survival of mice and reduced gross stomach weight (Figure 8, D–F). Histological staining showed reduced submucosal invasion and improved gastric glandular organization as evidenced from IHC of α-SMA, GKN2, and PGC (Figure 8G and Supplemental Figures 15 and 16). Together, these results suggest that *Kmt2c/d* loss–mediated gastric cancer in TPCD confers sensitivity to mTORC1 inhibition.

### Combination of rapamycin and anti–PD-1 suppresses TPCD cell growth in vivo.

To evaluate the combinatorial effects of mTORC1 inhibition and immunotherapy in vivo, we grafted TPCD tumor cells into C57BL/6 mice and treated them with vehicle, rapamycin, anti–PD-1, or the combination. Monotherapy with either rapamycin or anti–PD-1 effectively suppressed tumor initiation, reducing tumor formation rate from 8/8 (vehicle) to 6/8 (anti–PD-1) and 3/8 (rapamycin) (Figure 8, H and I). The combination further inhibited tumorigenesis, with a 1/8 tumor formation rate by day 28 postgrafting (Figure 8H). Consistent with the secretion function of stomach mucosa (42), fluid secretion was observed in some tumors in the control group (Figure 8H). After puncturing the tumors and aspirating the fluid, a significant reduction in tumor weight was identified (Figure 8J). Treatment with rapamycin led to decreased p-S6 (Ser235/236) level, and anti–PD-1 treatment increased CD8^+^ T cell infiltration, confirming the efficacy of these treatments (Supplemental Figure 17A). Additionally, rapamycin treatment improved histological differentiation, though no change in CDX2 expression was observed (Supplemental Figure 17A). In an independent allograft experiment with delayed treatment initiation until tumors reached 80 mm^3^, anti–PD-1 monotherapy showed mild efficacy in suppressing tumor growth, whereas the combination demonstrated substantial synergy compared with either monotherapy (Supplemental Figure 17, B–D). Together, our findings highlight the potential of combining mTORC1 inhibition and immune checkpoint blockade for *KMT2C/D*-deficient STAD treatment.

## Discussion

The identification of driver mutations in MSI tumors is difficult because of a large number of passenger mutations, particularly in large genes. TCGA specifically excluded MSI tumors from analysis of recurrently mutated genes (3, 20, 21). However, hotspot mutations in oncogenes are well-known drivers in MSI cancer, such as *KRAS*, *PIK3CA*, and *BRAF^V600E^*. A comparison of KMT family of histone methyltransferase genes between gastric and colorectal cancer found *KMT2C* to be selectively mutated in MSI gastric cancers, and mutated samples lost all protein staining by IHC (43). A pan-TCGA analysis of MSI that specifically analyzed frameshift events at microsatellites found cancer-specific preferences, including higher frequency of events at microsatellites in *KMT2C* and *KMT2D* in gastric cancer (44). Our analysis of TCGA data suggests the positive enrichment of *KMT2C* and *KMT2D* LOF mutations in STAD, as *KMT2C* and *KMT2D* LOF are much less common in MSI cancers in other cancer types and other similarly large genes do not harbor as many LOF mutations in STAD.

These data led us to study the in vivo role of *Kmt2c* and *Kmt2d* in GEMMs. Our data showed that *Kmt2c/d* loss promotes gastric cancer initiation and drives marked molecular and phenotypic changes, including impaired cellular differentiation, enhanced antigen presentation, and reduced protein synthesis. Notably, in the small and large intestines, these changes were not observed, recapitulating human pathology. These findings provide critical insights into the role of *Kmt2c/d* as key regulators of gastric epithelial homeostasis and tumorigenesis. Despite the high prevalence of *KMT2C/D* LOF mutations in STAD, knockout of *Kmt2c* or *Kmt2d* alone was insufficient to induce cancer initiation. A second oncogenic mutation, such as *Pten* loss, is required to fully drive tumorigenesis, consistent with reports in lung cancer and urothelial cancer (10, 12). The inability of *Kmt2c/d* loss alone to initiate cancer may be attributed to the decreased new protein synthesis, implicating the potential tumor-promoting role of PI3K and MAPK signaling in the context of *Kmt2c/d* deficiency.

Precancerous lesions are considered precursors in gastric cancer initiation (45, 46). Consistent with scRNA-seq data from human stomach IM tissues (45), loss of *Kmt2c/d* led to dysplasia and expansion of cells with mixed pit, neck, and stem cell features. These observations were accompanied by the mosaic expression of Alcian blue–positive mucin and positive staining of CDX2 in TCD and TPCD mice, suggesting the emergence of intestinal differentiation and the high relevance of our GEMMs to clinical observations. Notably, recent sequencing studies have identified *KMT2C* and *KMT2D* nonsynonymous mutations in IM and further identified *KMT2D* as one of the driver genes in IM progression (46, 47). In the TPCD stomach cancer model, we observed reduced expression of key gastric lineage markers, indicating a shift toward a less differentiated state. Additionally, this model expresses high levels of CLDN18, an FDA-approved therapeutic target in gastric cancer (48, 49), and may be a useful model for mechanistic and preclinical studies. These molecular and histological alterations closely mirror the progression of human STAD, reinforcing the relevance of our findings to human disease.

Our data reveal upregulated MHC-I molecule expression and enhanced antigen presentation in *Kmt2c/d*-deficient cells, suggesting a potential vulnerability to immune-based therapies. This is further supported by the increased sensitivity of *Kmt2c/d*-deficient cells to OT1 CD8^+^ T cells in vitro and to anti–PD-1 treatment in vivo, consistent with observations in liver and bladder cancer (10, 39). Our data are consistent with clinical observations of improved response to immune checkpoint blockade (ICB) in tumors with *KMT2C* or *KMT2D* mutations (10, 50). While ICB has been approved for most gastric cancers and is highly active in the MSI subgroup enriched for *KMT2C/D* LOF mutations (51), our findings may point to the utility of ICB in the *KMT2D*-mutant and *KMT2C/D*-mutant CIN subtype STADs. We further found that loss of *Kmt2c/d* led to decreased protein translation both in vivo and in organoids, leading to sensitization to mTORC1 inhibitors in organoids in vitro and in allograft tumors in vivo. Everolimus (RAD001) has been studied in a phase III trial in previously treated advanced gastric cancer. In the overall population, there was a small but significant improvement in progression-free survival but only a trend for overall survival (52). Our data suggest that loss of *KMT2C/D* may be a biomarker for those who may benefit from mTORC1 pathway inhibition. Our findings highlight the potential therapeutic strategy of combining mTORC1 inhibition with ICB for *KMT2D*-deficient and *KMT2C/D*-deficient STAD. Rapamycin is a potent immunosuppressant and combination with anti–PD-1 may seem counterintuitive. However, several recent studies have shown that rapamycin and JAK inhibitors paradoxically improve efficacy of anti–PD-1 therapy (53, 54). Moreover, rapamycin has been shown to suppress growth of *Arid1a*-deficient stomach cancer cells (7), suggesting a broader applicability of combining mTORC1 inhibitors and ICB in STAD treatment.

Our study has several limitations. *Tmprss2-CreER^T2^* is active in several endoderm-derived epithelial lineages, and tamoxifen injection induces robust recombination in the stomach, intestines, bladder, and luminal prostate epithelial cells. This limits long-term studies and the ability to evaluate metastasis in the GEMM. We have used organoids and allografts from GEMM mice to orthogonally validate our findings. Methods to localize recombination may overcome some limitations (24).

## Methods

### Sex as a biological variable.

Both male and female mice were used in this study. Sex was not considered as a primary biological variable, as no sex-dependent differences were observed in the experimental readouts. Data from both sexes were therefore analyzed together unless otherwise indicated.

### Mouse studies.

Mice were maintained under specific pathogen–free conditions, with a 12-hour light/12-hour dark cycle (lights on/off at 6 am/pm), controlled temperature (18°C–24°C) and humidity (40%–60%), and access to chow and sterilized water ad libitum. The following genetically engineered mouse strains were used: *Tmprss2-CreER^T2^-IRES-nlsEGFP* [*Tmprss2^tm1.1(cre/ERT2)Ychen^*, MGI:5911389], *Pten^fl^* (*Pten^tm2.1Ppp^*, MGI:2679886), *Kmt2c^fl^* (exon 3 flanked by *LoxP* sites), *Kmt2d^fl^* (exons 50–51 flanked by *LoxP* sites), and *Rosa26-CAG-LSL-EYFP* [*B6.Cg-Gt(ROSA)26Sor^tm3(CAG-EYFP)Hze^*, stock no. 007903, The Jackson Laboratory] (10). Primers for genotyping are listed in Supplemental Table 2. To induce *Tmprss2-CreER^T2^* activity, 2 doses of tamoxifen (Toronto Research Chemicals, T006000, 3 mg per dose in corn oil) were administered intraperitoneally to 8- to 12-week-old mice with interval of 48 hours.

To test the effect of rapamycin on stomach cancer progression in GEMMs, we administered tamoxifen to *Tmprss2-CreER^T2^ Pten^fl/fl^ Kmt2c^fl/fl^ Kmt2d^fl^* (TPCD) mice to induce gene knockout. Three days after the first dose of tamoxifen administration, mice were treated with either vehicle (5% ethanol, 5% PEG-400, 5% Tween-80 in PBS, 200 μL per injection) or rapamycin (HY-10219, MedChemExpress, 5 mg/kg, once daily, 5 days a week, 200 μL per injection) via intraperitoneal injection.

To assess the in vivo responses to rapamycin and ICB, we grafted 5 million TPCD cells into the mammary fat pad of 6- to 8-week-old female mice (C57BL/6J, stock no. 000664, The Jackson Laboratory). At 2 or 3 weeks after grafting, mice were treated with vehicle, rapamycin, IgG control (BE0089, clone 2A3, Bio X Cell, 8 mg/kg, twice a week, 200 μL in PBS), or anti–PD-1 (BE0146, clone RMP1-14, Bio X Cell, 8 mg/kg, twice a week, 200 μL per injection). Tumor sizes were measured twice a week using a digital caliper and calculated using the formula:

(Equation 1)
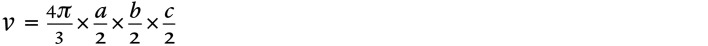


where a, b, and c represent the length, width, and thickness of the tumor, respectively.

### scRNA-seq and analysis.

Stomachs were collected from TY (*n* = 2 mice), TP (*n* = 3 mice), TCD (*n* = 3 mice), and TPCD (*n* = 2 mice) groups 3 weeks after tamoxifen administration. Stomach mucosae were stripped using tweezers and washed with cold PBS. After mincing with a scalpel, the mucosal tissues were digested for 30 minutes with TrypLE (12605010, Gibco), followed by 45 minutes with collagenase/hyaluronidase (07912, STEMCELL Technologies). Viable cells were sorted as DAPI-negative using a BD FACSymphony S6 Cell Sorter. For each mouse, 10,000 cells were processed for encapsulation and library preparation (Chromium Next GEM Single Cell 3’ GEM, Library and Gel Bead Kit, 10x Genomics). In each sample, 200 million reads were acquired on a NovaSeq S4 flow cell platform (Illumina).

scRNA-seq data were analyzed as previously described (10, 55). Briefly, sequencing reads were mapped to the mouse genome (GRCm38) using the Cell Ranger (7.0.0) software (10x Genomics). Downstream analysis and figure plotting were processed using Scanpy (1.9.8) (56). Cells were removed if they expressed fewer than 100 unique genes, fewer than 2,000 total counts, more than 40,000 total counts, or greater than 20% mitochondrial reads. Genes detected in fewer than 20 cells and all mitochondrial genes were excluded from subsequent analyses. Combining samples from all cohorts yielded a count matrix of 47,777 cells by 20,618 genes, with a median of 9,037 counts and a median of 2,575 genes per cell. The count matrix was normalized by log_2_(10K+1) to identify the top 2,000 highly variable genes. The count matrix was further scaled to a mean of 0 and a standard deviation of 1 for principal component analysis (PCA), UMAP dimensionality reduction, and Leiden clustering (57). PCA was performed on the 1,000 most variable genes, and the top 50 principal components retained 41% of the variance. Cell types were determined using a combination of markers genes identified in prior literature and the web-based tool PanglaoDB (https://panglaodb.se/) (30).

Differentially expressed genes among each group were compared using Scanpy (1.9.8) (56). For GSEA comparing genotypes of gastric cells using mouse scRNA-seq data, we pooled all gastric epithelial cells of each genotype to generate pseudo-bulk expression data. We generated the ranked gene list using the difference of pseudo-bulk log_2_ expression between genotypes. For GSEA comparing *KMT2C/D* LOF samples and other samples, we calculated the mean log_2_ expression of all genes in samples with *KMT2C* or *KMT2D* LOF mutations (nonsense or splice site) and in the remaining samples. We generated the ranked list using the difference of mean log_2_ expressions. GSEA was performed on the ranked gene list using the JAVA GSEA 4.1.0 program, with curated gene sets (C2, C8) and the Hallmark gene set (H) from the Molecular Signatures Database v7.4 using gene set permutation.

The processed scRNA-seq data from human precancerous and cancerous stomachs were downloaded from National Center for Biotechnology Information (NCBI) Gene Expression Omnibus (GEO) GSE134520 and GSE183904. We used the same filtration parameters as the original studies. Harmony (0.0.10) algorithm was used for the integrated analyses (38).

### Mouse stomach cell culture.

Mouse stomach epithelial cells were isolated as described above. Cells were cultured in growth factor–reduced Matrigel matrix (356231, Corning) using organoid culture medium. The medium formulation is as follows: advanced DMEM/F12 (12634010, Gibco), B27 supplement (2% v/v, 17504044, Gibco), FBS (5% v/v, FB-11, Omega Scientific), Noggin conditioned medium (10% v/v), R-Spondin conditioned medium (10% v/v), Wnt-3A conditioned medium (50%, v/v), HEPES (2 mM, pH 7.4), EGF (50 ng/mL, AF-100-15, PeproTech), FGF10 (200 ng/mL, AF-100-26, PeproTech), Y-27632 (10 μM, S1049, Selleckchem), A83-01 (0.5 μM, S7692, Selleckchem), N-acetyl-l-cysteine (1.25 mM, A9165, MilliporeSigma), SB202190 (10 μM, S1077, Selleckchem), nicotinamide (10 mM, N0636, MilliporeSigma), gastrin (10 nM, G9145, MilliporeSigma), primocin (100 μg/mL, ant-pm-2, InvivoGen), penicillin-streptomycin (1% v/v, 15140122, Gibco), l-glutamine (1% v/v, 25030081, Gibco), and GlutaMAX (1% v/v, 35050061, Gibco). Successful deletions were validated using primers amplifying the floxed alleles. Primers for genotyping are listed in Supplemental Table 1.

To assess cellular responses to mTORC1 and mTORC2 inhibitors in Matrigel culture, TP and TPCD cells were mixed 1:2 with Matrigel and seeded at 500 cells per blob (50 μL final volume). Cells were treated starting from day 2 for 10 days, with the medium and inhibitors refreshed every 4 days. At the endpoint, images of organoids were captured using a Nikon ECLIPSE Ti2 inverted microscope. Organoids were digested with TrypLE for 30 minutes at 37°C. After centrifugation (200*g*), cells were lysed with CellTiter-Glo luminescent reagent (G9243, Promega) and measured using the GLOMAX 96-microplate luminometer (Promega).

To culture stomach epithelial cells under 2-dimensional conditions, plates or dishes were coated with collagen I (A1048301, Gibco, 1:100 dilution in cold-sterilized water) for 1 hour at room temperature (28). Cells were cultured using the same stomach organoid medium. To test dose responses to PI3K/AKT, mTORC1, and mTORC2 inhibitors (BYL-719, S2814; AZD8186, S7694; ipatasertib, S2808; rapamycin, S1039; RAD001, S1120; INK-128, S2811; Selleck Chemicals), 1,000 TP and TPCD cells were seeded in a precoated, 96-well plate (100 μL final volume). Cells were treated starting from day 2 for 5 days, and cell viability was measured using the CellTiter-Glo luminescent reagent on a GLOMAX 96-microplate luminometer.

### Bulk RNA-seq.

Mouse stomach epithelial TY, TP, and TPCD cells were treated with DMSO or rapamycin (10 nM) for 24 hours. Total RNA was extracted suing TRIzol reagent (Invitrogen). RNA-seq libraries were prepared using poly-A capture protocol. Next-generation sequencing was performed by the Memorial Sloan Kettering Cancer Center (MSKCC) Integrated Genomics Operation (IGO) on Illumina NovaSeq 6000 with paired-end 100 bp for 30–40 million reads. The sequencing data were mapped to the mouse genome (GRCm38, mm10) using STAR (2.7.10b) (58).

### ChIP-seq.

In TP and TPCD cells, we performed ChIP-seq of H3K4me1 and H3K4me3. In each experiment, we used 1 μg antibodies against H3K4me1 (5326, clone D1A9, Cell Signaling Technology) or H3K4me3 (130060, EpiCypher). The library preparation and next-generation sequencing were performed by the MSKCC IGO core facility using Illumina NovaSeq 6000 with paired-end 100 bp for 30 million to 40 million reads. The sequencing data were processed for adapter trimming and aligned to the mouse genome (GRCm38, mm10) using bowtie2 (2.4.5) (59). MACS3 (3.0.0) was used to call broad and narrow peaks from both replicates with *P* < 0.05 (60). We quantified the read counts at the merged H3K4me1 and H3K4me3 peaks using featureCounts (v2.0.1) (61). Due to overall decrease of H3K4me1 level in TPCD cells, the H3K4me1 ChIP-seq was normalized using spike-in Drosophila genome (53083 and 61686, Active Motif). Heatmaps and aggregation plots were generated using deepTools (3.5.1) (62).

### OVA antigen presentation and OT1 CD8^+^ T cell killing assay.

OT1 CD8^+^ T cells were negatively isolated by depletion of magnetically labeled cells (130-104-075, Miltenyi Biotec) from the spleen of OT-1 mice [C57BL/6-Tg(TcraTcrb)1100Mjb/J, 003831, The Jackson Laboratory]. Plates were precoated with anti-CD3 antibody (2 μg/mL in PBS, 100302, clone 145-2C11, BioLegend) overnight at 4°C. Naive CD8^+^ T cells were cultured with plate-bound anti-CD3 antibody and soluble anti-CD28 (2 μg/mL, 102102, clone 37.51, BioLegend) antibody for 48 hours (63). T cell culture medium was prepared using RPMI-1640, supplemented with FBS (10% v/v), penicillin-streptomycin (1% v/v), l-glutamine (1% v/v), 2-mercaptoethanol (1:1,000, 21985-023, Gibco), and interleukin-2 (10 ng/mL, 402-ML, R&D Systems). CD8^+^ T cells were then expanded for 2 more days in T cell culture medium.

To induce presentation of OT-1–binding antigen SIINFEKL in stomach cells, we used the lentiviral vector pLVX-puro-cOVA (135073, Addgene) to exogenously express chicken OVA protein. We detected the expression of MHC-I components (H-2Kb/Db-APC, 114614, clone 28-8-6, BioLegend) and the presentation of SIINFEKL (H-2Kb–bound SIINFEKL-APC, 141606, clone 25-D1.16, BioLegend) using a BD LSRFortessa instrument. In the coculture killing assay, 50,000 TY-OVA, TP-OVA, and TPCD-OVA cells were seeded in collagen I–coated, 12-well plates. On the second day, medium was replaced with T cell culture medium containing mouse IFN-γ (10 ng/mL, 485-MI, R&D Systems). Twenty-four hours later, OT1 CD8^+^ T cells were mixed with tumor cells at ratios of 1:1, 2:1, and 5:1. After overnight incubation, floating T cells and dead stomach cells were decanted and washed twice with PBS. The remaining cells were measured using CellTiter-Glo luminescent reagent on a GLOMAX 96-microplate luminometer.

### Histological analysis.

Mouse stomach, intestine, and colon tissues were dissected and opened longitudinally. After washing in cold PBS, tissues were fixed in 4% paraformaldehyde, dehydrated, and embedded in paraffin. Paraffin embedding and sectioning were performed by Histoserv Inc. IHC was conducted using a Ventana automatic stainer. The following primary antibodies were used in this study: H3K4me1 (5326, clone D1A9, Cell Signaling Technology, 1:100), PGC (ab180709, Abcam,1:2,000), MUC5AC (MA1-21907, clone 45M1, Thermo Fisher Scientific, 1:1,000), ATP4A (D031-3, MBL Life Science, 1:1,000), Ki-67 (ab16667, clone SP6, Abcam, 1:100), CDX2 (MA5-14494, clone EPR2764Y, Thermo Fisher Scientific, 1:500), α-SMA (ab5694, Abcam, 1:1,000), B2M (HPA006361, MilliporeSigma, 1:500), CD8α (98941, clone D4W2Z, Cell Signaling Technology, 1:100), p-S6 Ser235/236 (2211, Cell Signaling Technology, 1:200), PTEN (9188, clone D4.3, Cell Signaling Technology, 1:100), E-cadherin (3195, clone 24E10, Cell Signaling Technology, 1:100), p-AKT Thr308 (13038, clone D25E6, Cell Signaling Technology, 1:100), and p-AKT Ser473 (4060, clone D9E, Cell Signaling Technology, 1:100). Both H&E and IHC slides were scanned using a Mirax digital slide scanner. The thickness of stomach mucosa was measured using QuPath 0.4.4.

IF staining was performed using primary antibodies against EYFP (2956, clone D5.1, Cell Signaling Technology, 1:100), H3K4me1 (5326, clone D1A9, Cell Signaling Technology, 1:100), ATP4A (D031-3, MBL Life Science, 1:500), and puromycin (MABE343, clone 12D10, MilliporeSigma, 1:100). Secondary antibodies conjugated with Alexa Fluor 488 (A11008, Thermo Fisher Scientific, 1:400) or Alexa Fluor 555 (A21424, Thermo Fisher Scientific, 1:400) were used in this study. The lectin GS-II Alexa Fluor 647 (L32451, Thermo Fisher Scientific, 1:500) was applied along with secondary antibodies. Fluorescent images were captured using a Leica TCS SP5 inverted confocal microscope.

Alcian blue staining was performed using paraffin sections. The Alcian blue staining solution was prepared by dissolving 1 g of Alcian blue (8GX) (A5268, Millipore Sigma) in 100 mL of 3% acetic acid at pH 2.5. Nuclear fast red staining solution was prepared by dissolving 1 g of nuclear fast red (60700, Millipore Sigma) and 50 g of aluminum sulfate (227617, Millipore Sigma) in 1 L of water. After dewaxing, sections were permeabilized with 0.5% Triton-X 100 for 10 minutes, stained with Alcian blue solution for 30 minutes, and counterstained with nuclear fast red solution for 5 minutes. Images were scanned using a Mirax digital slide scanner.

BaseScope was performed using Leica Bond RX as previously described (10). Fresh sections were stained with probes for *Kmt2c* (#1285828-C1, ACD Bio) and *Kmt2d* (#1285848-C1, ACD Bio). BaseScope LS Reagent Kit-RED was used to visualize the signal (#323600, ACD Bio). Sections were scanned using Mirax digital slide scanner.

### Human STAD dataset analysis.

To analyze gene expression differences between *KMT2C/D*-LOF samples and the remaining samples, we used the cBioPortal annotation of the TCGA STAD (https://www.cbioportal.org/study/summary?id=stad_tcga_pub, TCGA STAD PanCancer https://www.cbioportal.org/study/summary?id=stad_tcga_pan_can_atlas_2018), TCGA COAD (https://www.cbioportal.org/study/summary?id=coadread_tcga_pub), and TCGA UCEC datasets (https://www.cbioportal.org/study/summary?id=ucec_tcga_pub). TCGA STAD PanCancer is a subset including 145 new samples acquired after initial publication of 295 samples. Analysis and OncoPrint were performed on cBioPortal. Comparisons were made using all samples (*n* = 295) or nonhypermutated samples (*n* = 226) from the 2014 TCGA STAD dataset and independently from the new samples in the TCGA STAD PanCancer dataset. GSEA was performed on the ranked gene list using the JAVA GSEA 4.1.0 program, with curated gene sets (C2, C8) and the Hallmark gene set (H) from the Molecular Signatures Database v7.4. Expression of gastric lineage genes in human normal stomach and STAD samples were extracted using GEPIA2 (http://gepia2.cancer-pku.cn/#analysis).

### Western blot.

Stomach epithelial cells were cultured under 2-dimensional conditions and treated with DMSO, rapamycin (S1039, Selleck Chemicals), RAD-001 (S1120, Selleck Chemicals), INK-128 (S2811, Selleck Chemicals), BYL-719 (S2814, Selleck Chemicals), AZD8186 (S7694, Selleck Chemicals), or ipatasertib (S2898, Selleck Chemicals) for 1 hour. Cells were lysed in RIPA buffer and quantified using the BCA method. Primary antibodies against β-actin (4970, clone 13E5, Cell Signaling Technology, 1:5,000), p70 S6 kinase (9022, Cell Signaling Technology, 1:2,000), p-p70 S6 kinase Thr389 (9234, clone 108D2, Cell Signaling Technology, 1:1,000), p-4EBP1 Thr37/46 (2855, clone 236B4, Cell Signaling Technology, 1:2,000), 4EBP1 (9644, clone 53H11, Cell Signaling Technology, 1:2,000), p-AKT Ser473 (4060, clone D9E, Cell Signaling Technology, 1:2,000), p-AKT Thr308 (13038, clone D25E6, Cell Signaling Technology, 1:2,000), AKT (4691, clone C67E7, Cell Signaling Technology, 1:5,000), H3K4me1 (5326, clone D1A9, Cell Signaling Technology, 1:1,000), H3K4me3 (9751, clone C42D8, Cell Signaling Technology, 1:2,000), H3 (ab18521, abcam, 1:10,000), KMT2A (14689, clone D2M7U, Cell Signaling Technology, 1:1,000), and KMT2B (47097, clone E3M1V, Cell Signaling Technology, 1:1,000) were applied. After incubation with HRP-conjugated secondary antibody (111-035-144, Jackson ImmunoResearch, 1:20,000), membranes were developed with enhanced chemiluminescence Western blotting substrate (32106, Thermo Fisher Scientific). Images were taken using an Amersham ImageQuant 800 biomolecular imager.

### Puromycin incorporation assay.

To detect new protein synthesis, we performed the puromycin incorporation assay. For the in vivo assay, mice were treated with an intraperitoneal injection of puromycin (200 μL per mouse, 2.5 mM, HY-B1743, MedChemExpress) 1 hour before tissue collection. Newly synthesized proteins were detected by IF staining using anti-puromycin antibody (MABE343, MilliporeSigma, 1:100). For the in vitro assay, cells were treated with 20 μM OPP for 30 minutes. The incorporated OPP was then detected using the Click-iT Plus OPP Alexa Fluor 647 Protein Synthesis Assay Kit (C10458, Thermo Fisher Scientific). Signals were detected using a BD LSRFortessa instrument.

### CRISPR/Cas9-mediated knockout.

In TP cells, *Kmt2c* and *Kmt2d* were knocked out using lentiCRISPRv2 plasmid with puromycin or hygromycin selection markers. Successful gene editing was validated using surveyor assay (M302, New England Biolabs).

Sequences of sgRNAs were sg*Kmt2c*: 5′–3′ GTGGGTCTTAATTATGCCCTGT; sg*Kmt2d*: 5′–3′ AGTACCTGGCTGTGCTAGATC.

### Quantitative PCR.

Total RNA was extracted from TP stomach cells transduced with sgControl, sg*Kmt2c*, and sg*Kmt2d* using the Total RNA Extraction Kit (R1034, Omega Bio-Tek). For cDNA synthesis, 1 μg of total RNA was reverse-transcribed using the high-capacity cDNA reverse transcription kit (4368814, Applied Biosystems). Quantitative PCR was subsequently performed using PowerUp SYBR Green Master Mix on a QuantStudio 7 Flex real-time PCR system. The primer sequences used in this study are provided in Supplemental Table 3.

### Statistics.

Statistical analysis was performed as detailed in the figure legends. Student’s *t* tests were 2-tailed, and 1-way or 2-way ANOVA was used where appropriate, as specified in the figure legends. A *P* value less than 0.05 was considered statistically significant. All results were successfully repeated with a minimum of 2 independent experiments. Plots were generated using GraphPad Prism 10.

### Study approval.

Mouse experiments were approved by the Institutional Animal Care and Use Committee of Memorial Sloan Kettering Cancer Center, New York, New York, USA.

### Data availability.

Raw sequencing data of scRNA-seq have been deposited to the NCBI GEO and are publicly available. The accession numbers are GSE292432, GSE316994, and GSE317003. The Supporting Data Values file is provided as a supplement to this manuscript. All other data supporting the finding of this study are available upon request from the corresponding authors.

## Author contributions

NW and YC contributed to conceptualization. NW, TZ, MRP, DMS, WHC, MNK, and KK contributed to investigation. DL, NW, and YC contributed to formal analysis. YB, LT, and YYJ contributed to histology review. YC and PC contributed to supervision. NW, TZ, DL, ML, YYJ, PC, and YC contributed to writing.

## Conflict of interest

PC has received personal honoraria/advisory boards/consulting fees from Deciphera, Exelixis, Zai Lab, Novartis, and Ningbo NewBay Medical Technology. PC has received institutional research funding from Pfizer/Array, Novartis, Deciphera, and Ningbo NewBay Medical Technology. YC has stock ownership and received royalties from ORIC Pharmaceuticals. YYJ has received personal advisory boards/consulting fees from AbbvVie, Alphasights, Amerisource Bergen, Ask-Gene Pharma, Inc., Arcus Biosciences, Astellas, AstraZeneca, Basilea Pharmaceutica, Bayer, Boehringer Ingelheim, Bristol Myers Squibb, Clinical Care Options, Daiichi-Sankyo, eChina Health, Ed Med, Resources (OncInfo), Eisai, Eli Lilly & Co., Geneos Therapeutics, GlaxoSmithKline, Guardant Health, Inc., H.C. Wainwright & Co., Health Advances, HMP Global, Imedex, Imugene, Inspirna, Lynx Health, Mashup Media LLC, Master Clinician Alliance, Merck, Merck Serono, Mersana Therapeutics, Michael J. Hennessy Associates, Oncology News, Paradigm Medical Communications, PeerMD, PeerView Institute, Pfizer, Physician’s Education Resource, LLC, Research to Practice, Sanofi Genzyme, Seagen, Silverback Therapeutics, Suzhou Liangyihui Network Technology Co., Ltd, Talem Health, TotalCME, and Zymeworks. YYJ has stock options from Inspirna and Veda Life Sciences, Inc.

## Funding support

This work is the result of NIH funding, in whole or in part, and is subject to the NIH Public Access Policy. Through acceptance of this federal funding, the NIH has been given a right to make the work publicly available in PubMed Central.

- National Institutes of Health (NIH), R01CA228216 (PC).
- National Institutes of Health (NIH), DP2CA174499 (PC).
- National Institutes of Health (NIH), P50CA217694 (PC).
- National Institutes of Health (NIH), U54CA224079 (YC).
- National Institutes of Health (NIH), P50CA092629 (YC).
- National Institutes of Health (NIH), P50CA221745 (YC).
- National Institutes of Health (NIH), R01CA193837 (YC).
- National Institutes of Health (NIH), U01CA224044 (YC).
- National Institutes of Health (NIH), R01CA208100 (YC).
- Samuel Waxman Cancer Research Foundation (YC).
- Gladstein Family Bladder Cancer Research Fund (YC).
- Geoffrey Beene Cancer Research Fund (YC).
- Geoffrey Beene Cancer Research Fund (PC).
- Department of Defense (DOD), W81XWH-15-1-0124 (PC).
- Francis Collins Scholar NTAP (PC).
- Cycle for Survival and Linn Family Discovery Fund (PC).

## Supplementary Material

Supplemental data

Unedited blot and gel images

Supplemental table 1

Supplemental table 2

Supplemental table 3

Supporting data values

## Version 1 05/12/2026

In-Press Preview

## Version 2 07/15/2026

Electronic publication
