# Supplementary material for: Loss of Kmt2c/d promotes gastric cancer and confers vulnerability to mTORC1 and PD-1 inhibition
Source: J Clin Invest. 2026 May 12;136(14):e194462. doi: 10.1172/JCI194462 (PMC13367975; doi:10.1172/JCI194462)
Supplement: Supplemental data [file jci-136-194462-s296.pdf]

Figure S1. Oncoprint of selected epigenetic genes in new STAD samples of the TCGA PanCancer atlas.

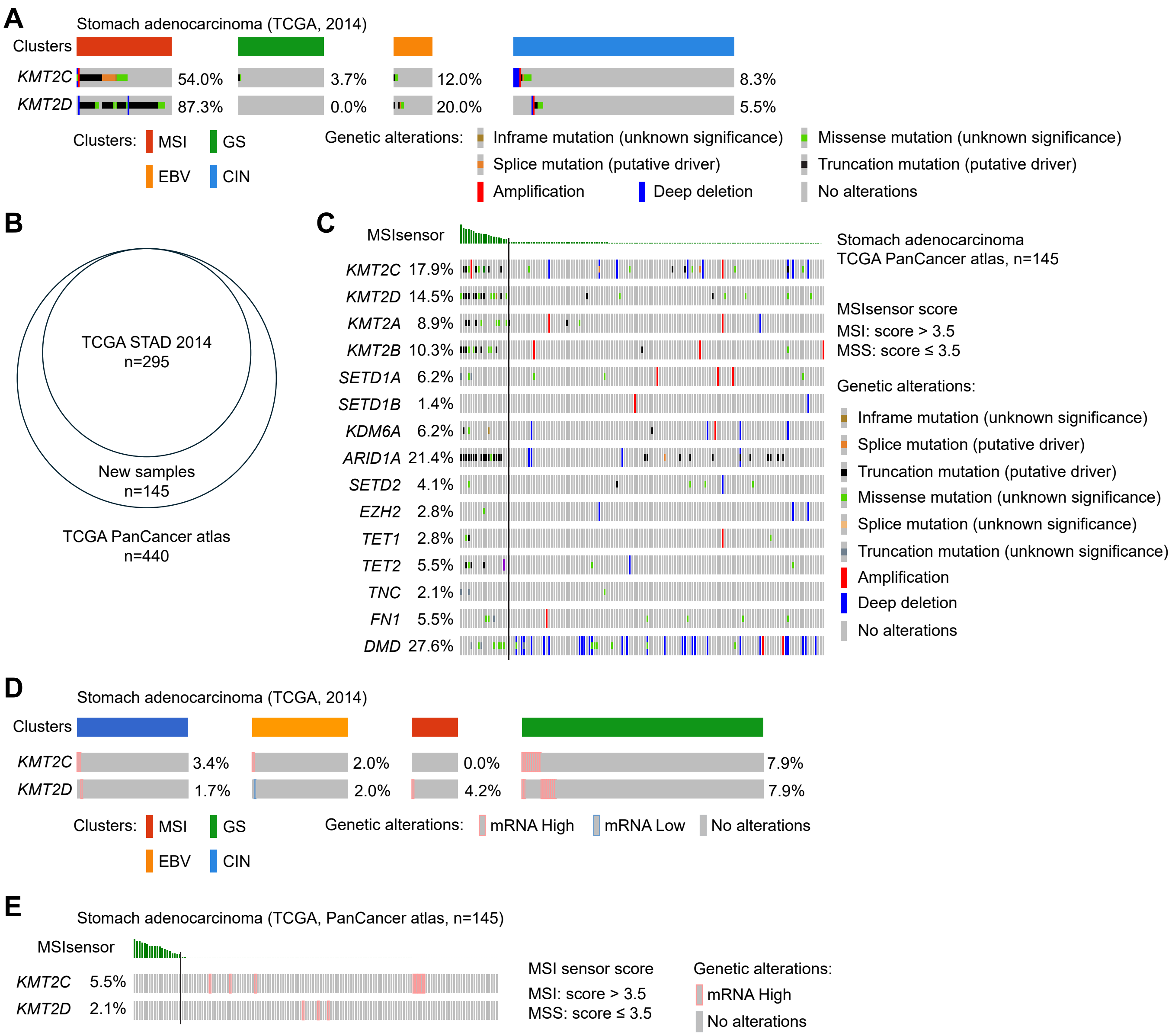

Figure S1. Oncoprint of selected epigenetic genes in new STAD samples of the TCGA PanCancer atlas.

A, Oncoprint of *KMT2C* and *KMT2D* in the TCGA stomach adenocarcinoma dataset.

B, Overlap of TCGA STAD 2014 dataset and TCGA PanCancer dataset. The 145 new samples were analyzed as an independent cohort.

C, OncoPrint of selected chromatin modifying genes and several other large genes in 145 new samples from the TCGA PanCancer atlas of stomach adenocarcinoma. Samples are sorted by MSIsensor score with a cutoff of 3.5 to indicate MSI samples.

D-E, OncoPrint of *KMT2C* and *KMT2D* gene expression in TCGA STAD dataset. The z-scores (RNA Seq V2 RSEM) were calculated relative to the expression distribution in diploid tumors for each gene. A z-score threshold of $\pm$ 2.0 was used to define genes as significantly upregulated or downregulated.

Figure S2. Validation of *Tmprss2-CreER^{T2}*-mediated gene knockout in GEMMs.

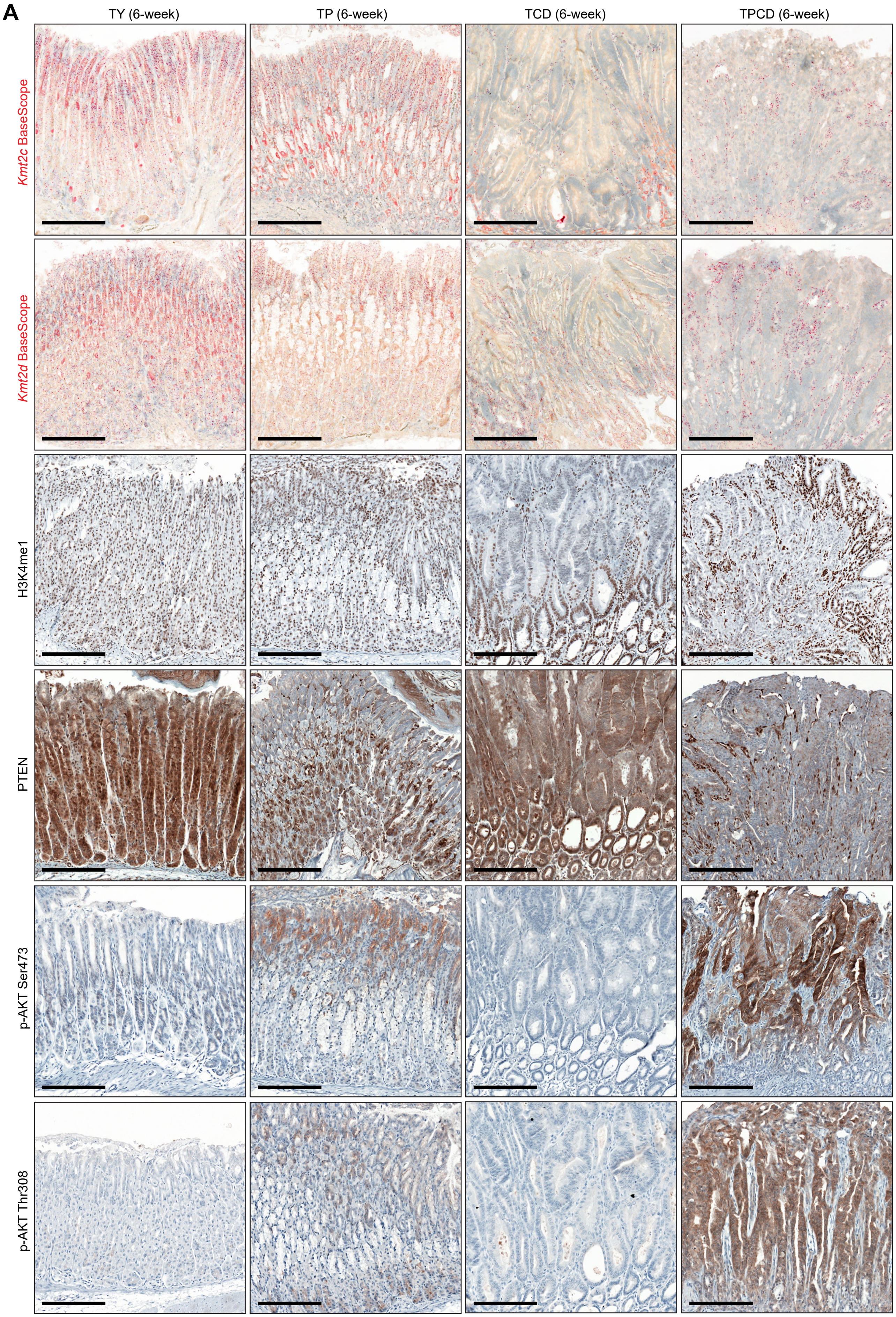

Figure S2. Validation of *Tmprss2-CreER*^{T2}-mediated gene knockout in GEMMs.

A, Top, representative BaseScope staining of stomach tissues with probes targeting *Kmt2c* or *Kmt2d* floxed exons. Bottom, representative IHC of H3K4me1, PTEN, p-AKT Ser473, and p-AKT Thr308 in stomachs. Tissues were collected 6 weeks post tamoxifen administration. Scale bar, 200 μm.

Figure S3. Depletion of both *Kmt2c* and *Kmt2d* is required for robust tumorigenesis.

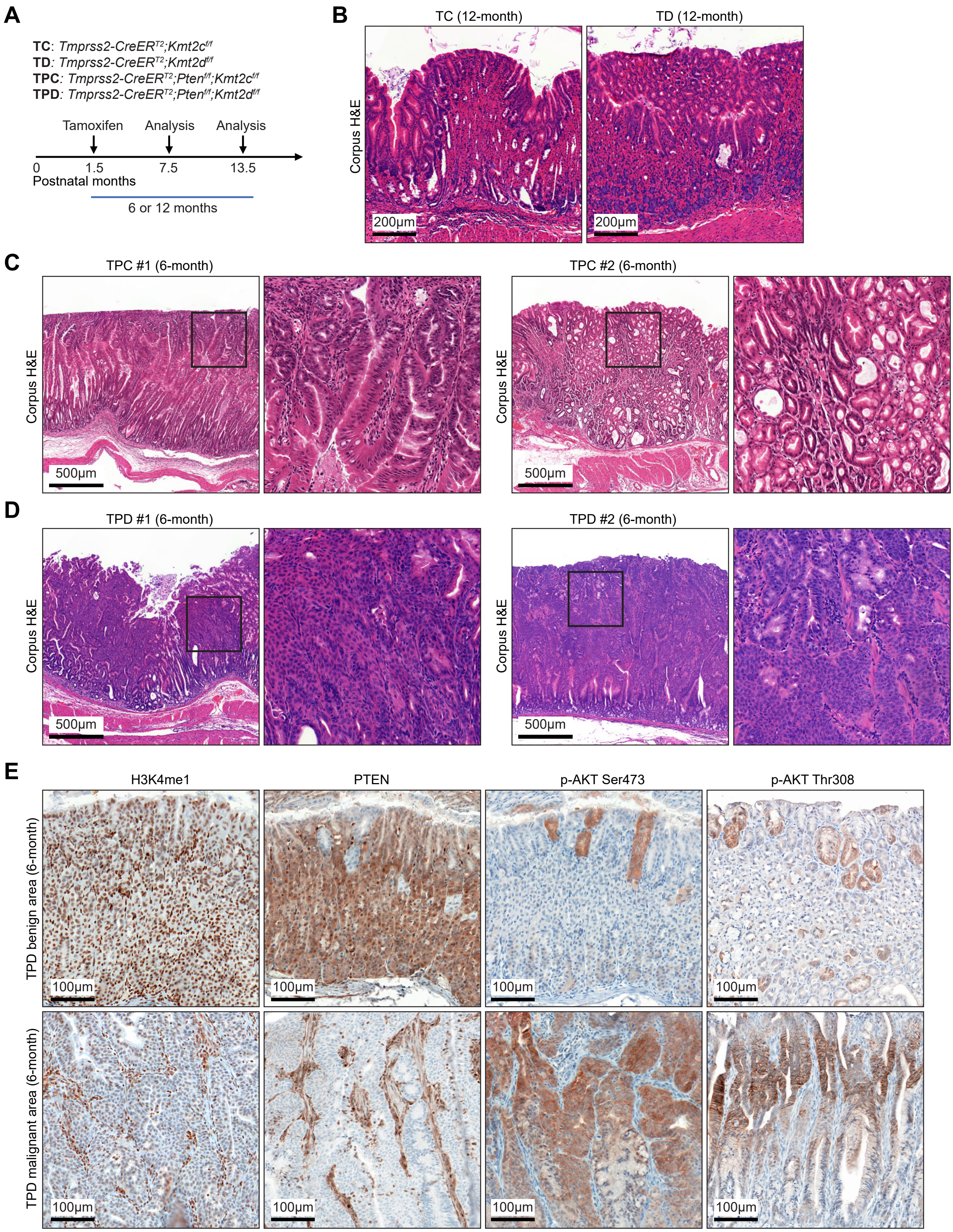

Figure S3. Depletion of both *Kmt2c* and *Kmt2d* is required for robust tumorigenesis.

A, Schematic of mouse models: two doses of tamoxifen (3 mg × 2) were injected intraperitoneally with a 48-hour interval.

B, Representative H&E staining of stomach tissues in TC and TD groups 12 months post tamoxifen administration. Scale bar, 200 μm.

C-D, Representative H&E staining of stomach tissues in TPC and TPD groups 6 months post tamoxifen administration. Scale bar, 500 μm.

E, Representative IHC of H3K4me1, PTEN, p-AKT Ser473, and p-AKT Thr308 in histologically benign and malignant areas of TPD stomach tissues 6 months post tamoxifen administration. Scale bar, 100 μm.

Figure S4. Representative H&E staining in mouse stomach tissues.

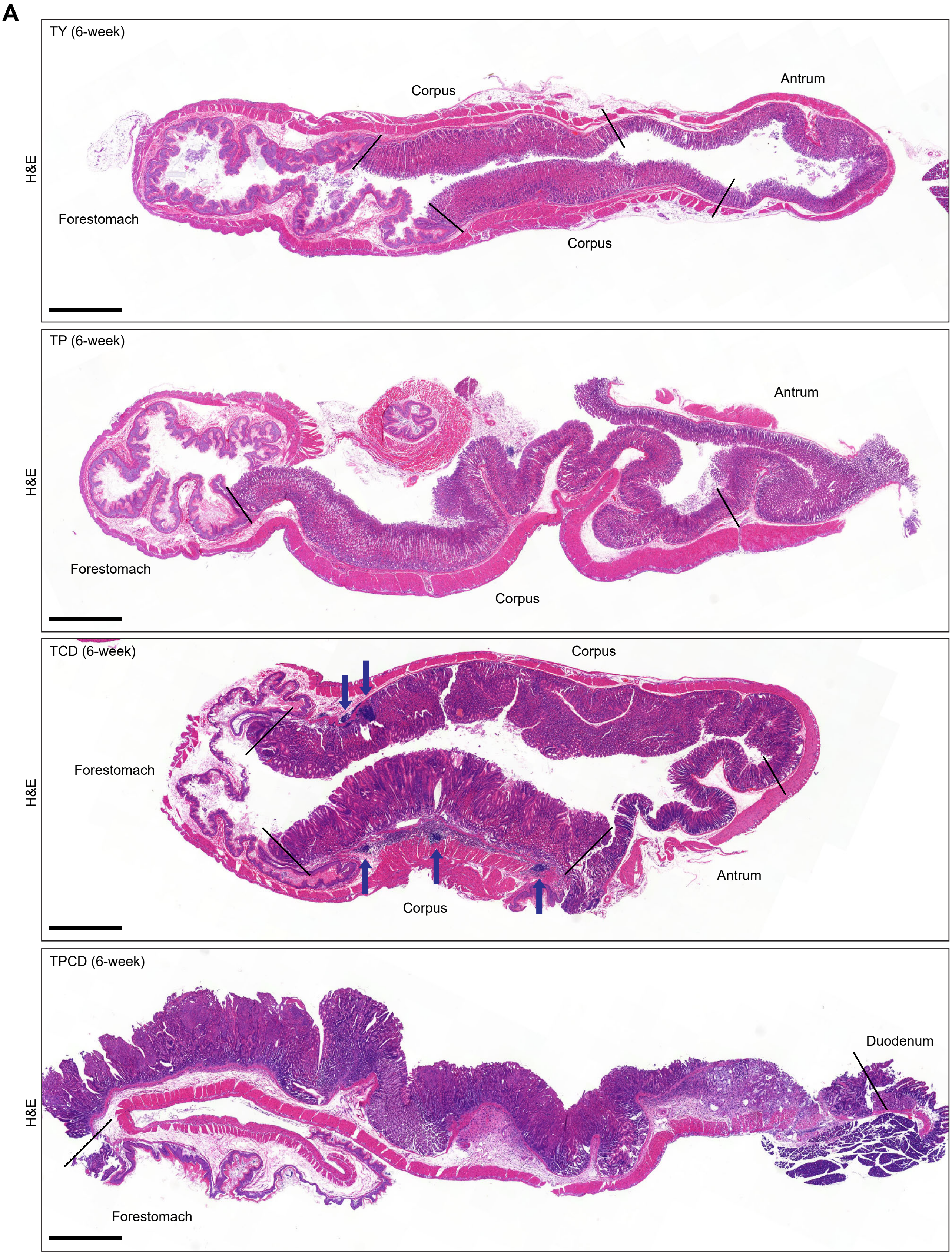

Figure S4. Representative H&E staining in mouse stomach tissues.

A, Representative H&E staining of TY, TP, TCD, and TPCD mice 6 weeks post tamoxifen administration. Lines are drawn to separate the antrum, corpus, and forestomach. Arrows in TCD group point to the secondary lymphoid structures.. Scale bar, 1 mm.

Figure S5. H&E staining showing muscle-invasive lesions in mouse stomach tissues.

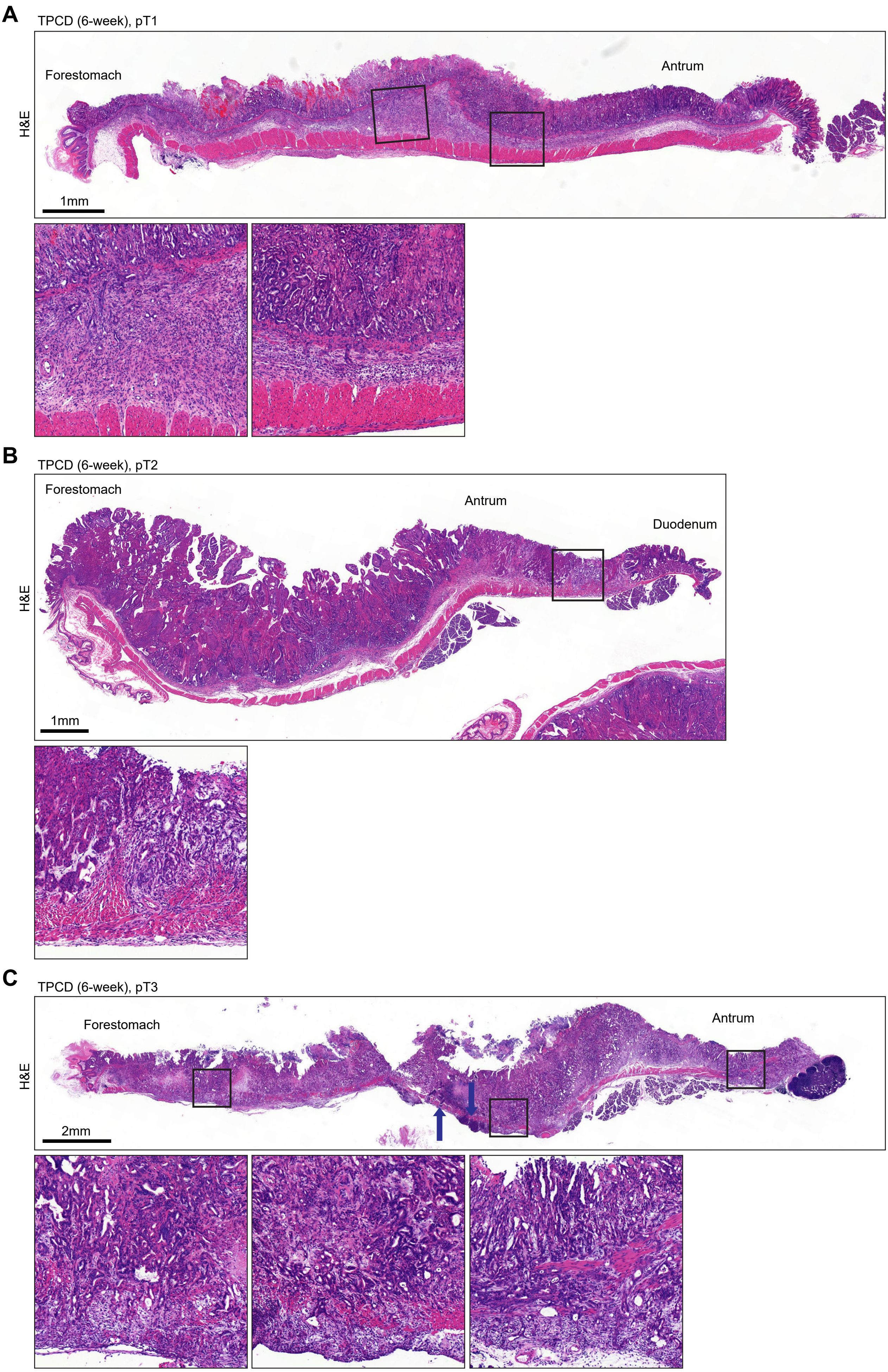

Figure S5. H&E staining showing muscle-invasive lesions in mouse stomach tissues.

A-C, Representative H&E staining of pT1 (invasion into the submucosa), pT2 (invasion into the muscularis), and pT3 (invasion into the serosa) stages in TPCD stomach tissues. Scale bar was indicated in the figures. Arrows in the pT3 group point to the secondary lymphoid structures.

Figure S6. IHC of E-cadherin in stomach draining lymph nodes from TPCD mice.

A

TPCD (6-week), pT3, greater curvature LN

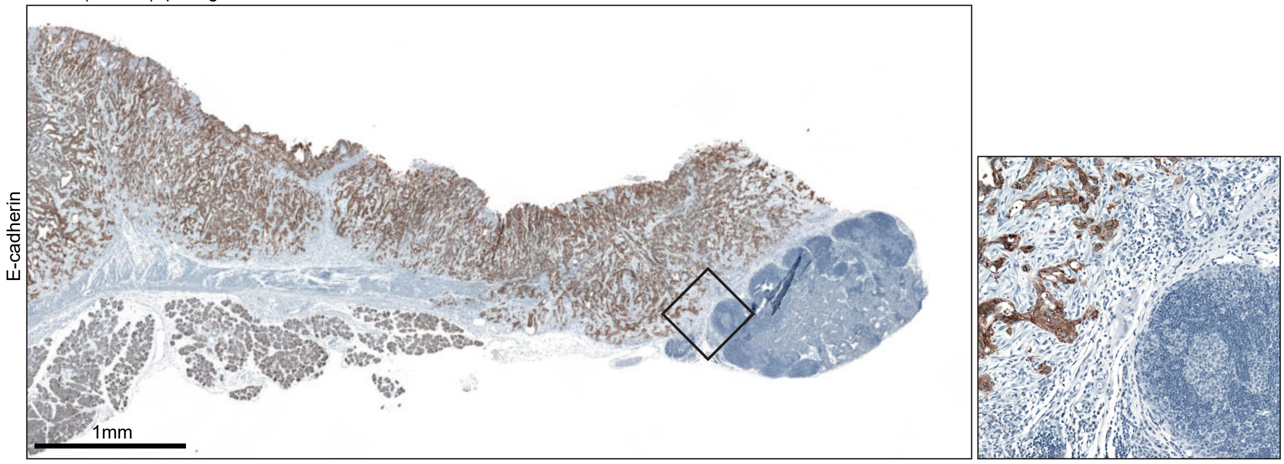

B

TPCD (3-week), pT1, celiac artery LN

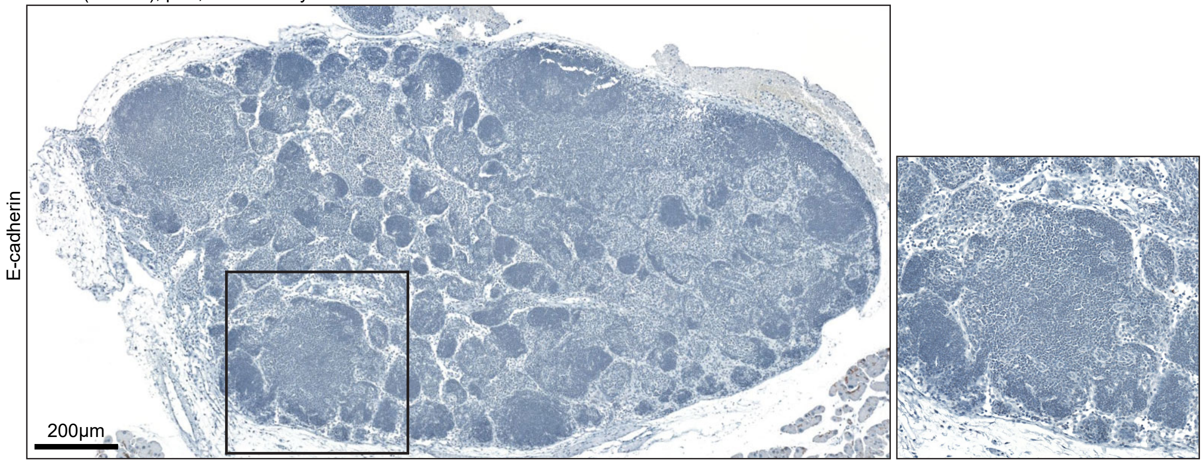

C

Days post tamoxifen administration	Pathological stage	Location of LNs	IHC of E-cadherin in LNs
23 days	pT1	Greater curvature LN	Negative
37 days	pT2	Greater curvature LN	Negative
37 days	pT3	Greater curvature LN	Negative
23 days	pT1	Celiac artery LN	Negative
23 days	pT2	Celiac artery LN	Negative
23 days	pT3	Celiac artery LN	Negative

Figure S6. IHC of E-cadherin in stomach draining lymph nodes from TPCD mice.

A, Representative E-cadherin IHC of a pT3 stomach cancer and an attached greater curvature lymph node. There were no E-cadherin positive cells in the lymph node to suggest metastasis.

B, Representative E-cadherin IHC of a celiac artery lymph node showing no cells with positive staining.

C, Summary of lymph node collection timepoint post tamoxifen administration.

Figure S7. Validation of gene knockout in the intestines of TPCD mice.

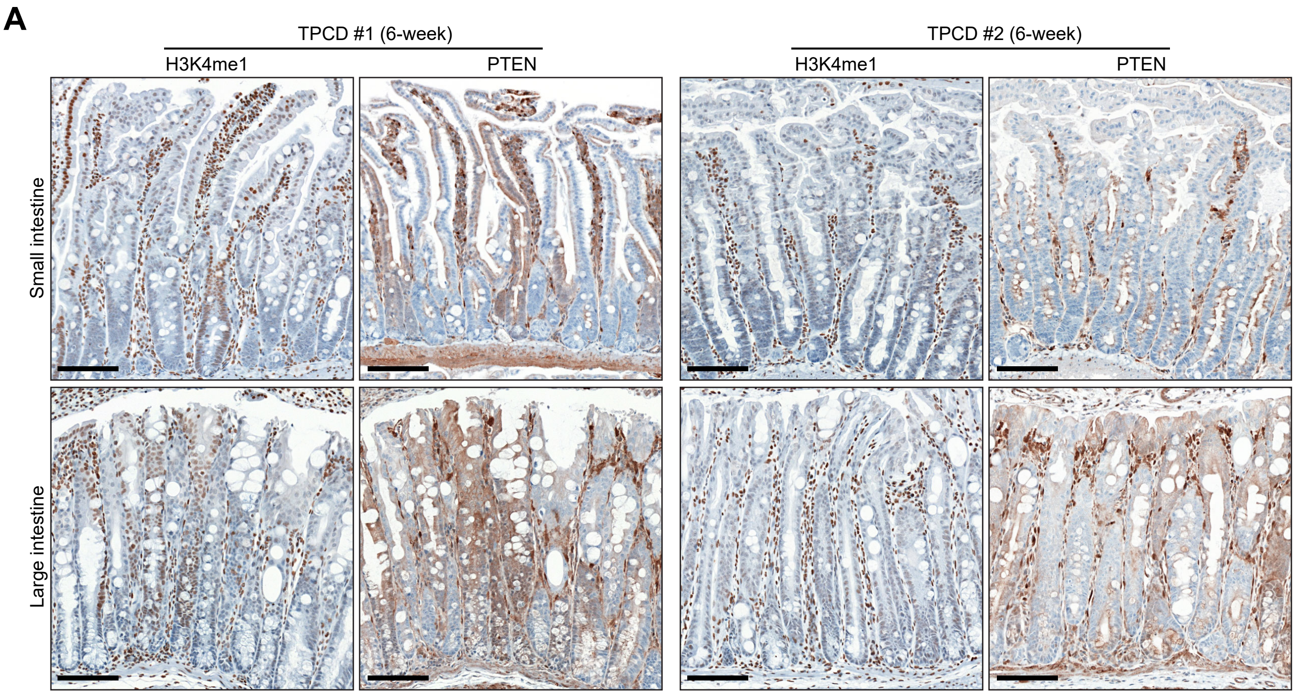

Figure S7. Validation of gene knockout in the intestines of TPCD mice.

A, Representative IHC of H3K4me1 and PTEN in the small and large intestines of two TPCD mice. Scale bar, 100 μm.

Figure S8. Characterization of cell clusters in scRNA-seq.

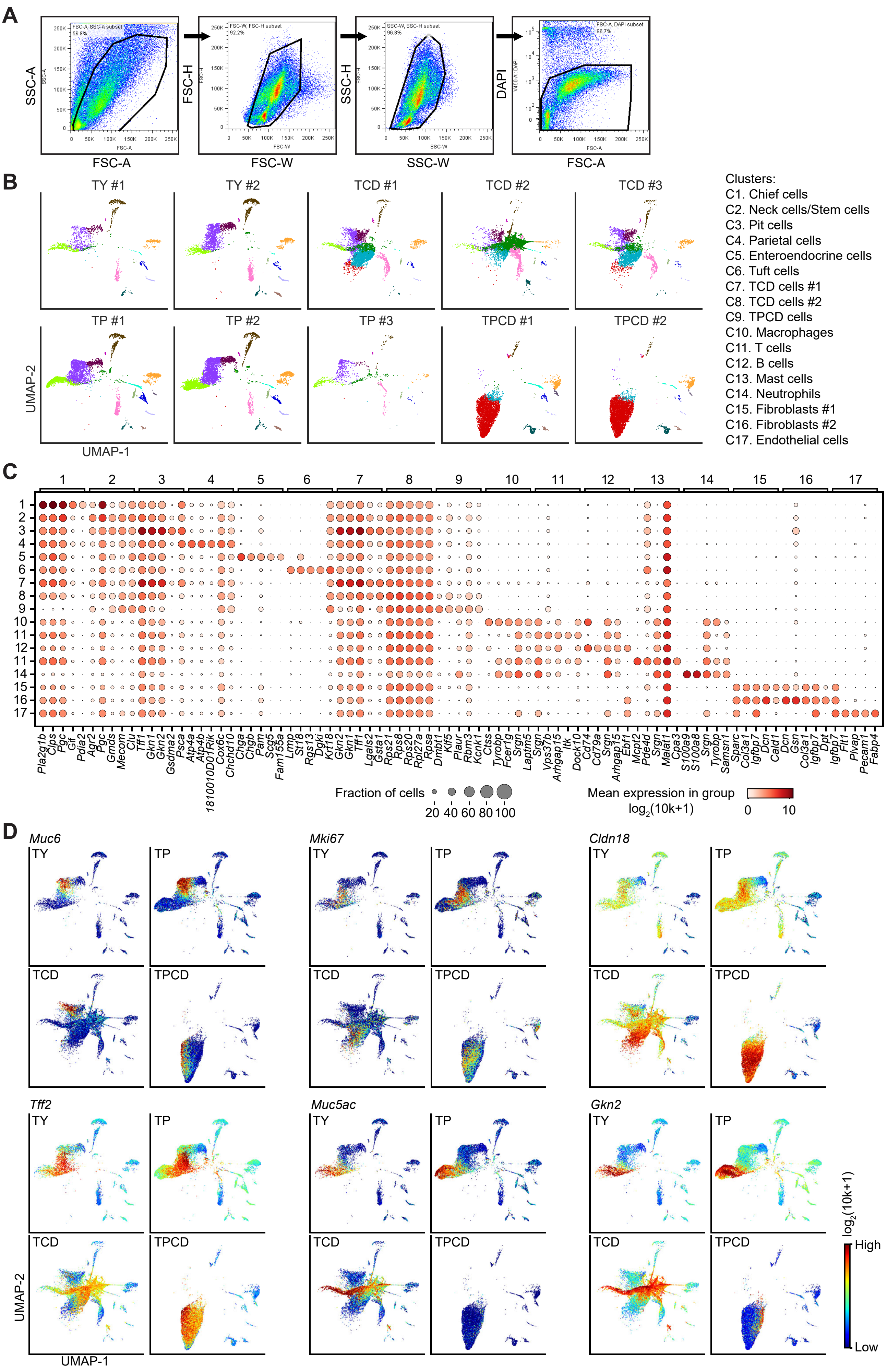

Figure S8. Characterization of cell clusters in scRNA-seq.

A, Fluorescence-activated cell sorting (FACS) strategy of DAPI-negative viable cells in dissociated stomach mucosa for scRNA-seq.

B, UMAP showing reproducibility of clusters in TY, TP, TCD, and TPCD mice.

C, Dot plot showing the expression of top 5 genes in each cluster.

D, Single cell expression level of the indicated gastric lineage markers in TY, TP, TCD, and TPCD mice shown on UMAP.

Figure S9. *Kmt2c/d* knockout impairs differentiation of stomach mucosa.

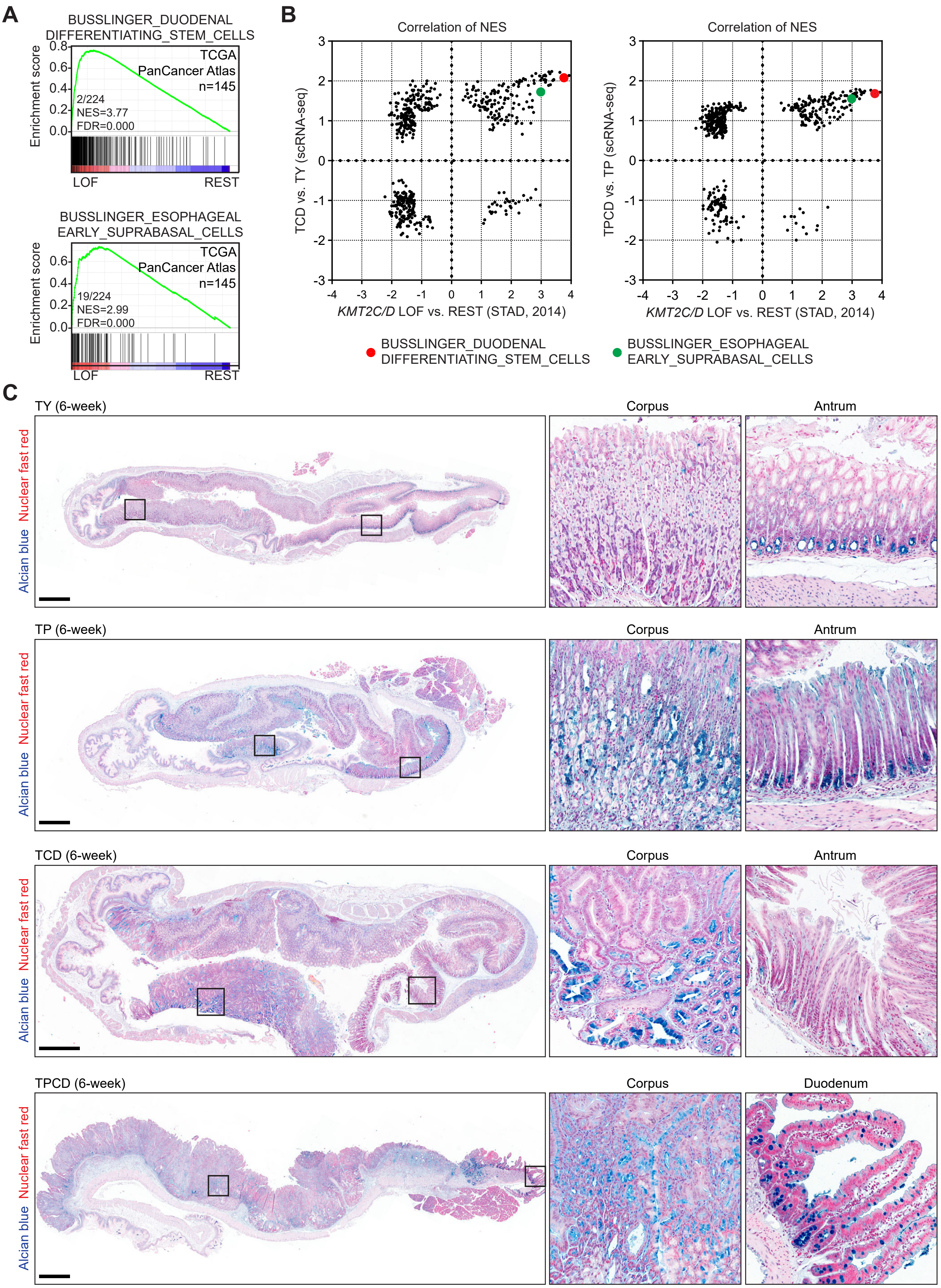

Figure S9. *Kmt2c/d* knockout impairs differentiation of stomach mucosa.

A, GSEA analysis in TCGA PanCancer atlas (n=145) showing positive enrichment of gene sets associated with duodenal and esophageal lineages in *KMT2C/D*-LOF STAD samples (n=16).

B, Correlation of GSEA enrichment scores between mouse stomach scRNA-seq and human TCGA STAD dataset.

C, Representative alcian blue staining in TY, TP, TCD, and TPCD stomach tissues. Nuclei were counterstained using nuclear fast red. Scale bar, 1 mm.

Figure S10. Knockout of *Kmt2c/d* upregulates the expression of MHC-I components in stomach cancer.

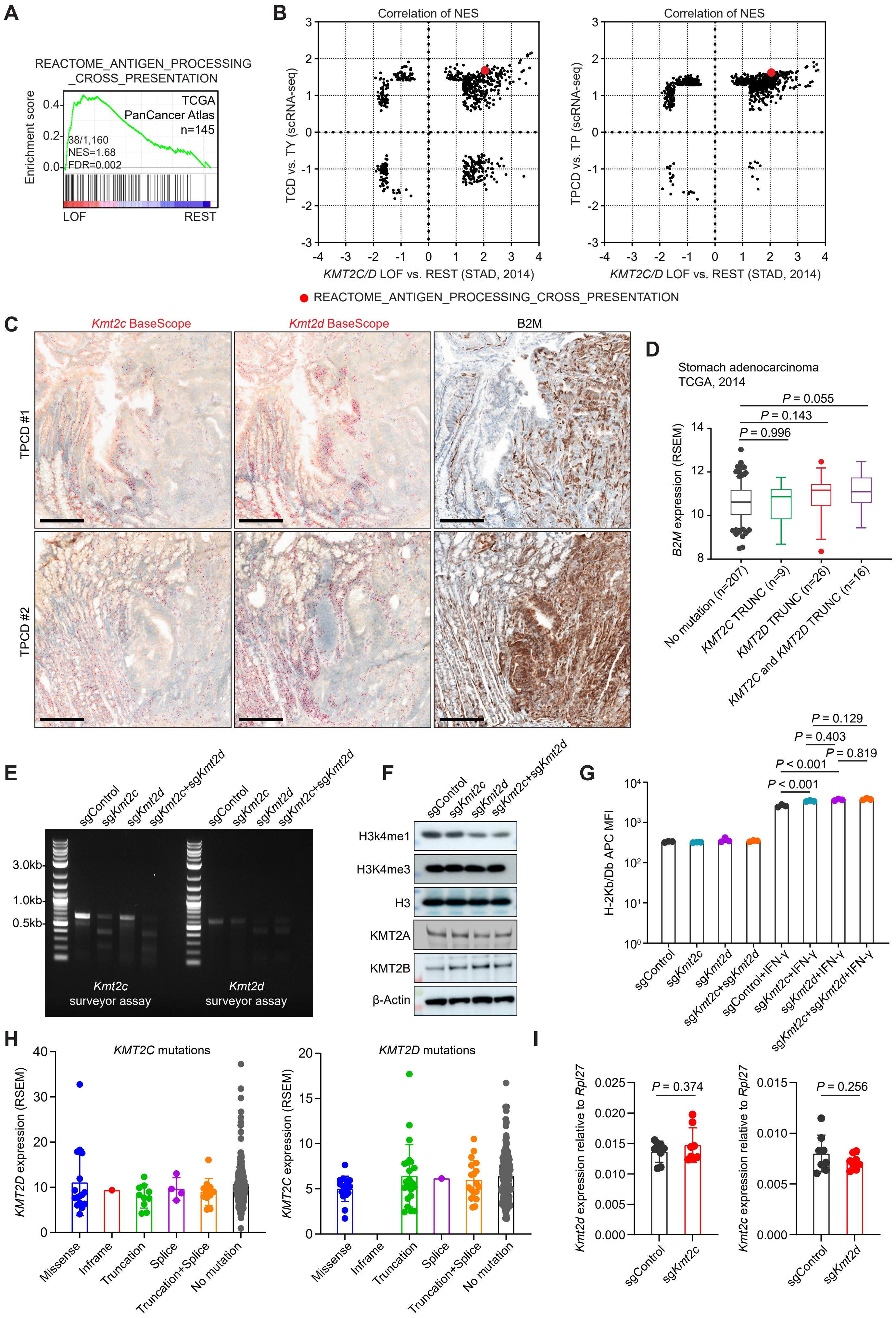

Figure S10. Knockout of *Kmt2c/d* upregulates the expression of MHC-I components in stomach cancer.

A, GSEA analysis in TCGA PanCancer atlas (n=145) showing positive enrichment of gene set associated with antigen presentation in *KMT2C/D*-LOF STAD samples (n=16).

B, Correlation of GSEA enrichment scores between mouse stomach scRNA-seq and human TCGA STAD dataset.

C, Representative BaseScope images of *Kmt2c* and *Kmt2d*, and representative IHC of B2M in TPCD stomach tissues. Note that *Kmt2c/d*-loss areas were negative for red dots, while *Kmt2c/d*-intact areas were positive for red dots. Scale bar, 200 μm.

D, Expression of *B2M* in *KMT2C* and/or *KMT2D* LOF mutation samples of the TCGA STAD 2014 dataset. The center line represents the median, the box limits represent the upper and lower quartiles and the minimum and maximum whiskers represent the 5th and 95th percentiles, respectively. Data were analyzed using one-way ANOVA followed by Dunnett's multiple comparisons test.

E, Surveyor assay validating the successful CRISPR editing of *Kmt2c* and *Kmt2d*.

F, Western blot analyses of H3K4me1, H3K4me3, H3, KMT2A, KMT2B, and β-Actin in TP cells infected with sgControl, sg*Kmt2c*, sg*Kmt2d*, and sg*Kmt2c*+sg*Kmt2d* CRISPR/Cas9 virus.

G, Mean fluorescence intensity of flow cytometry analysis of H-2Kb/Db in sgControl, sg*Kmt2c*, sg*Kmt2d*, and sg*Kmt2c*+sg*Kmt2d* cells. Data are shown on a logarithmic scale due to the wide dynamic range and right-skewed distribution of flow cytometry data, enabling appropriate visualization of fold changes. Data are presented as mean ± SD (n=3) and analyzed using two-way ANOVA followed by Tukey's multiple comparisons test on log_{10} normalized data.

H, Analyses of the TCGA STAD 2014 dataset showing no obvious impact of *KMT2C* and *KMT2D* expression after the loss of the other gene. Data are presented as mean ± SD.

I, Quantitative PCR analyses of *Kmt2c* and *Kmt2d* expression after CRISPR/Cas9 knockout of the other gene in gastric cells isolated from TP mice. Data were presented as mean ± SD (n=8) and analyzed using two-tailed t-test.

Figure S11. Validation of gene knockout in stomach organoids.

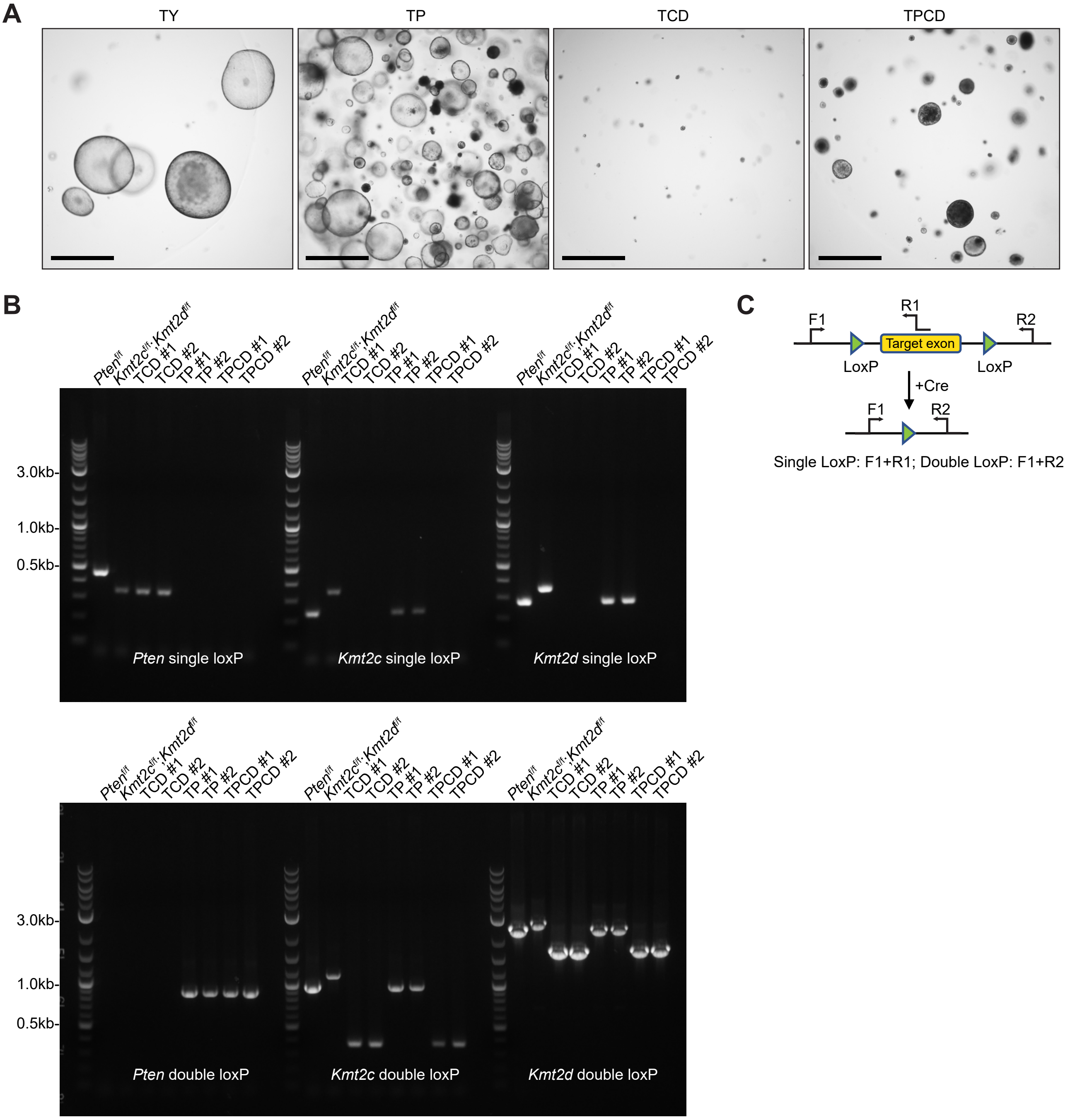

Figure S11. Validation of gene knockout in stomach organoids.

A, Representative bright-field images of organoids isolated from TY, TP, TCD, and TPCD groups after 4-hydroxytamoxifen treatment. Scale bar, 1 mm.

B, Genotyping of *Pten*, *Kmt2c*, and *Kmt2d* floxed alleles before and after 4-hydroxytamoxifen (0.2 μM, 24 hours) treatment.

C, Schematic of "Single LoxP" PCR that detects the intact Floxed allele and "Double LoxP" PCR that detects the recombined allele (or intact allele at much bigger size).

Figure S12. *Kmt2c/d* loss reduces new protein synthesis.

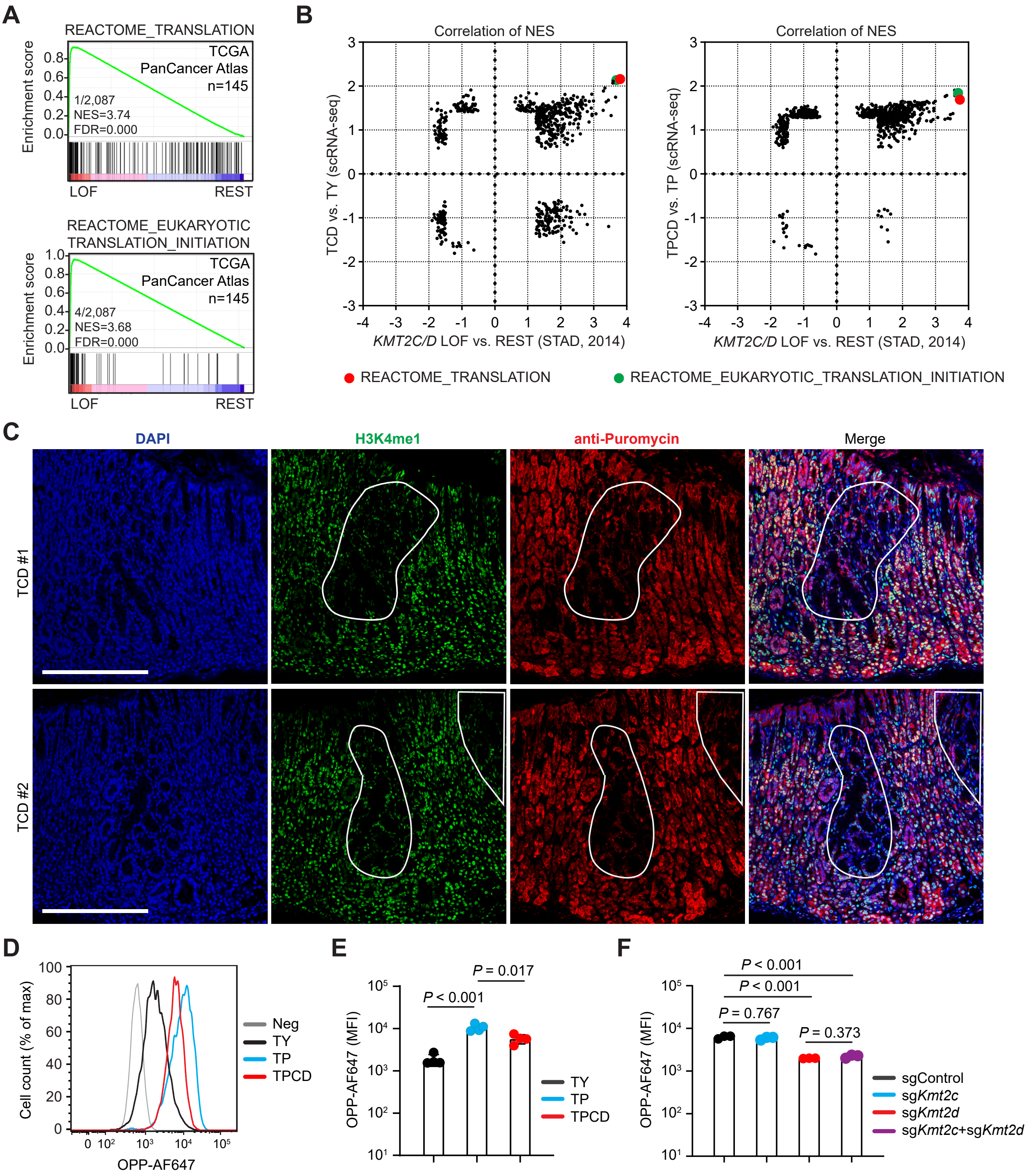

Figure S12. *Kmt2c/d* loss reduces new protein synthesis.

A, GSEA analyses in TCGA PanCancer atlas showing positive enrichment of gene sets associated with protein translation in *KMT2C/D*-LOF samples (n=16).

B, Correlation of GSEA enrichment scores between mouse stomach scRNA-seq and human TCGA STAD dataset.

C, Representative IF staining of H3K4me1 and puromycin in TCD mice treated with puromycin. Cells in the highlighted area exhibited weaker staining of H3K4me1 and puromycin. Nuclei were counterstained with DAPI. Scale bar, 200 μm.

D-E, Flow cytometry analysis of OPP incorporation in TY, TP, and TPCD stomach cells. Median fluorescent intensity values are shown on a logarithmic scale due to the wide dynamic range and right-skewed distribution of flow cytometry data, enabling appropriate visualization of fold changes. Data are presented as mean ± SD (n=4) and analyzed using one-way ANOVA followed by Sidak's multiple comparisons test on $\log_{10}$ normalized data.

F, Flow cytometry analysis of OPP incorporation in TP cells infected with sgControl, sg*Kmt2c*, sg*Kmt2d*, and sg*Kmt2c*+sg*Kmt2d* CRISPR/Cas9 virus. Median fluorescent intensity values are shown on a logarithmic scale due to the wide dynamic range and right-skewed distribution of flow cytometry data, enabling appropriate visualization of fold changes. Data are presented as mean ± SD (n=3) and analyzed using two-way ANOVA followed by Tukey's multiple comparisons teston $\log_{10}$ normalized data.

Figure S13. ChIP-seq of H3K4me1 and H3K4me3 in TP and TPCD cells.

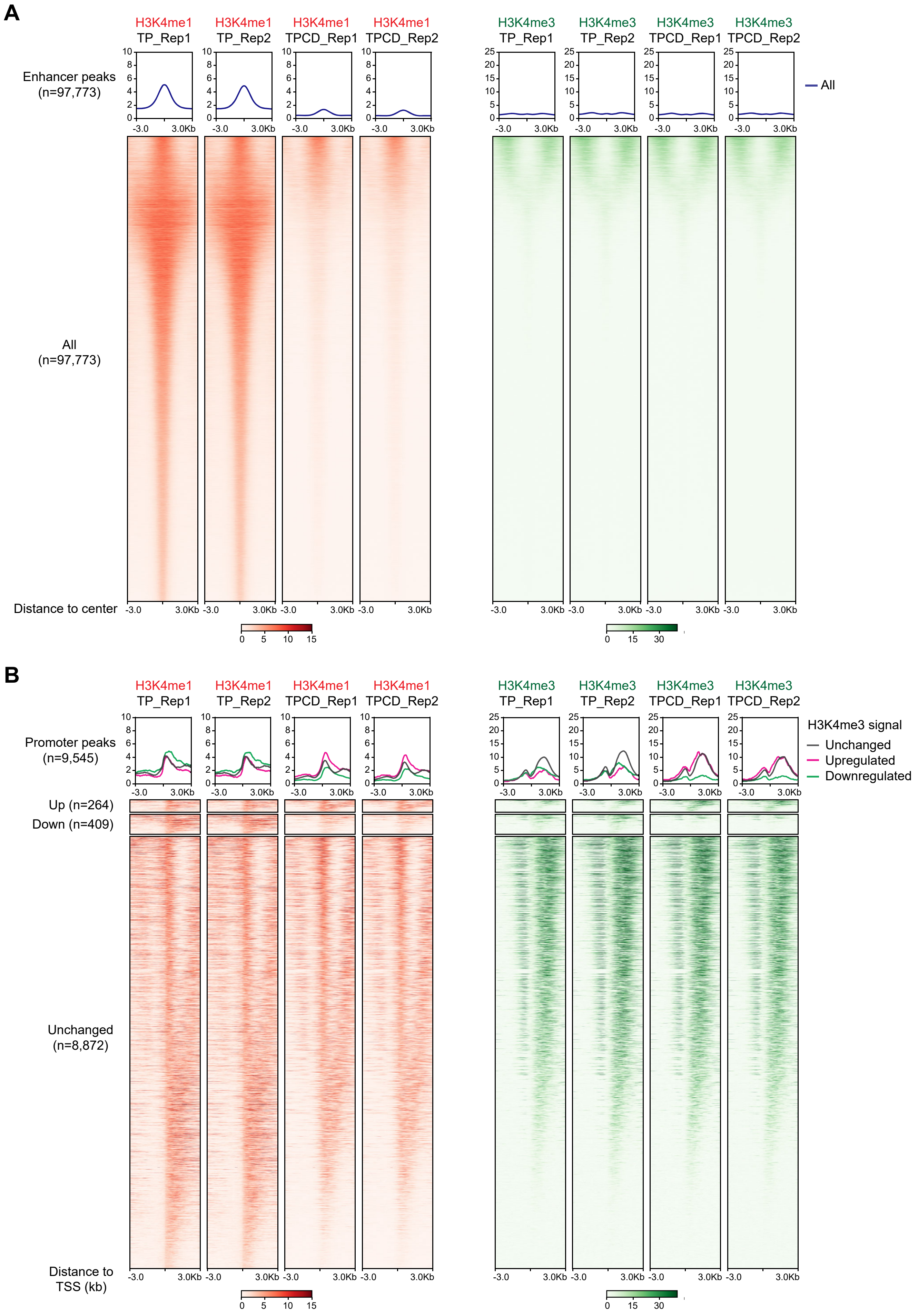

Figure S13. ChIP-seq of H3K4me1 and H3K4me3 in TP and TPCD cells.

A-B, ChIP-seq heatmap and profile showing the enrichment of H3K4me1 and H3K4me3 at enhancers and promoters. Enhancers are centered around peak center and promoters are centered around annotated TSS.

Figure S14. Drug sensitivity and immunoblot analyses of mTORC1 downstream signal with inhibitor treatment.

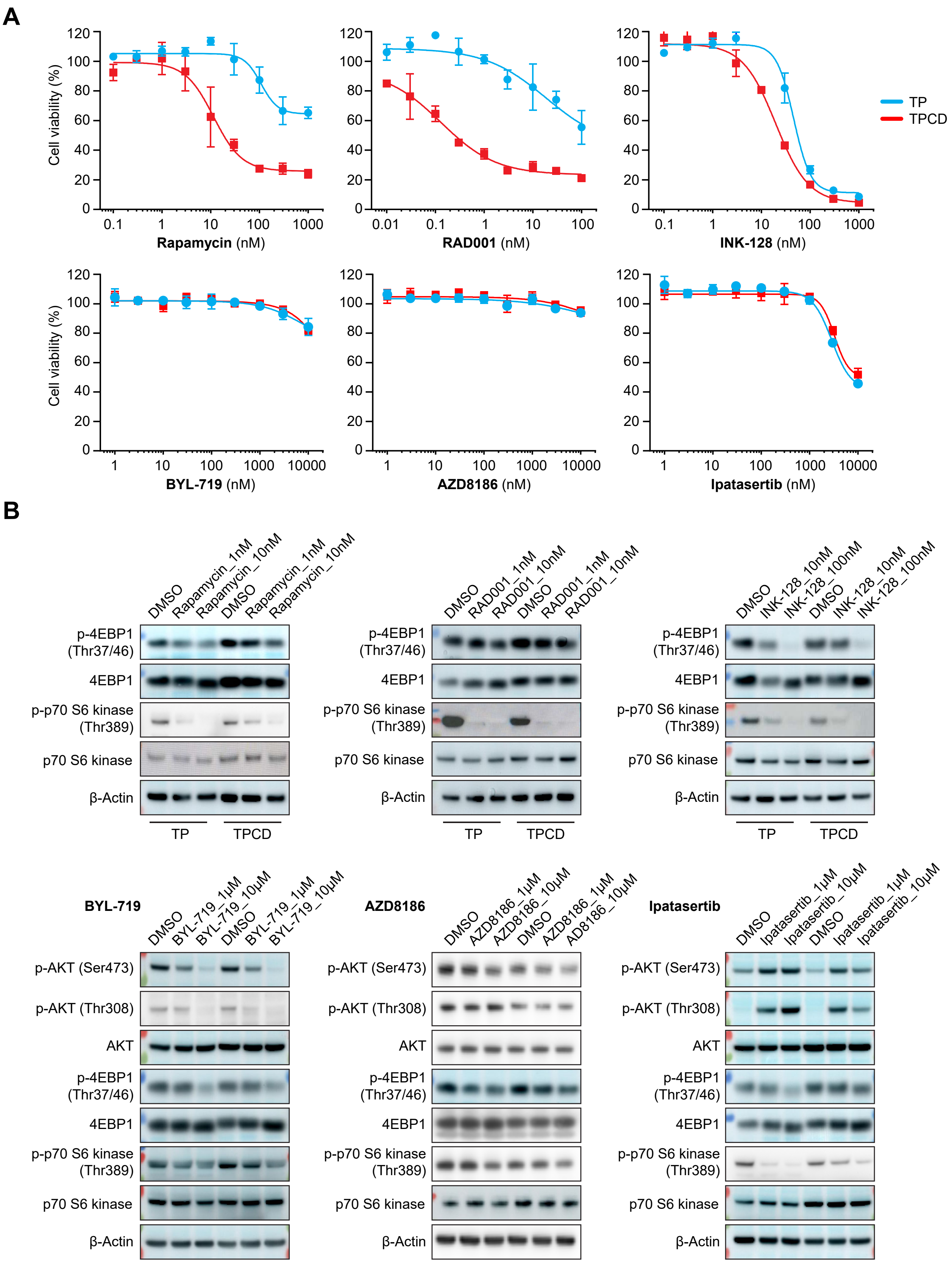

Figure S14. Drug sensitivity and immunoblot analyses of mTORC1 downstream signal with inhibitor treatment.

A, Cell viability following treatment of TP and TPCD cells with rapamycin, RAD-001, INK-128, BYL-719, AZD8186, or ipatasertib for 5 days. Cell viability was measured using CellTiter-Glo (n=3). Data are presented as Mean ± SD.

B, Western blot analysis showing decreased phosphorylation of 4EBP1 (Thr37/46) and p70 S6 kinase (Thr389) with PI3K-AKT-mTOR inhibitors treatment. TP and TPCD cells were treated for 1 h before sample collection.

Figure S15. Rapamycin treatment reduces muscle-invasive lesions in TPCD mice.

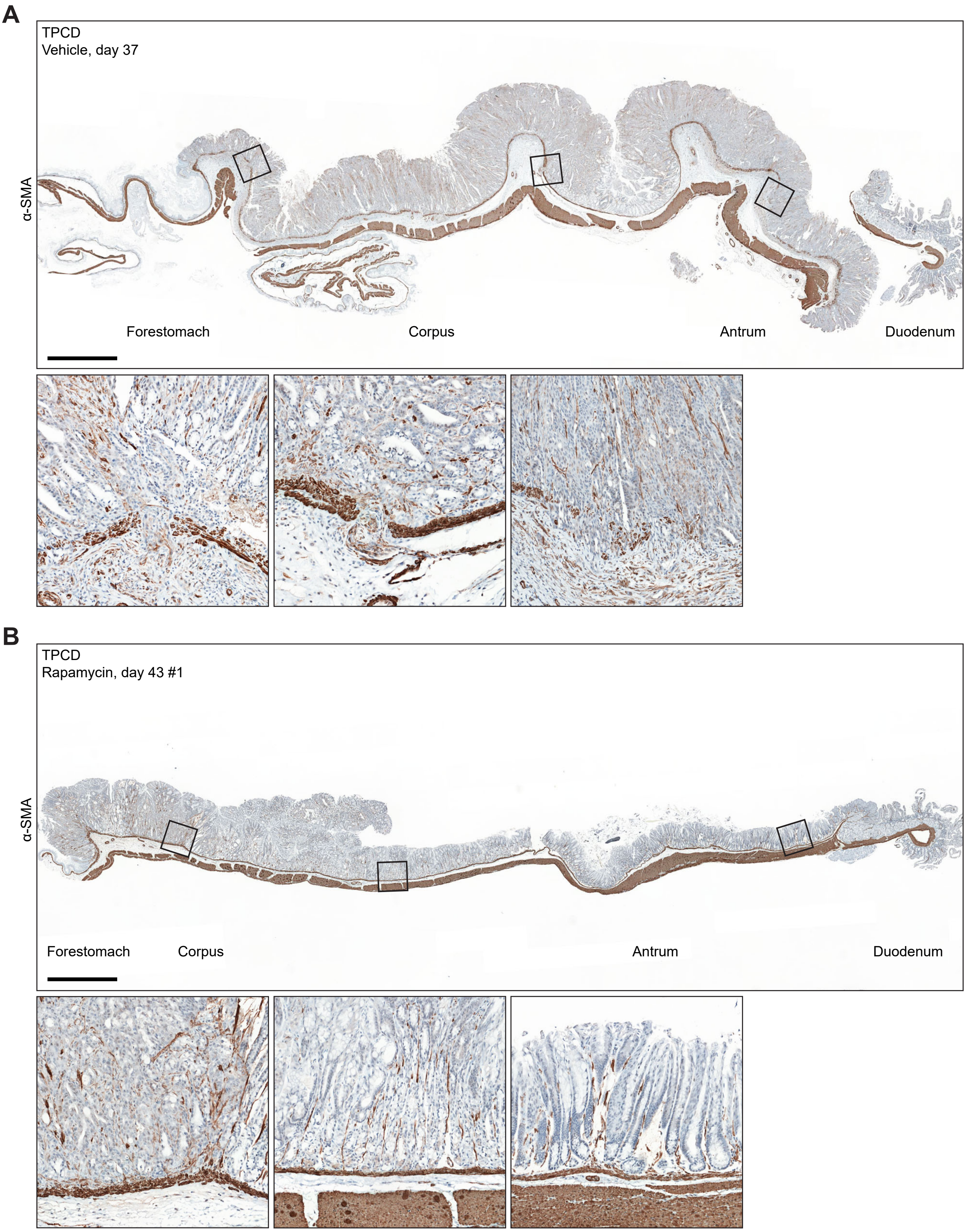

Figure S15. Rapamycin treatment reduces muscle-invasive lesions in TPCD mice.

A, Representative IHC of α-SMA in TPCD stomach tissues treated with vehicle or rapamycin.

Scale bar, 1 mm.

Figure S16. Rapamycin treatment rescues gastric differentiation in TPCD mice.

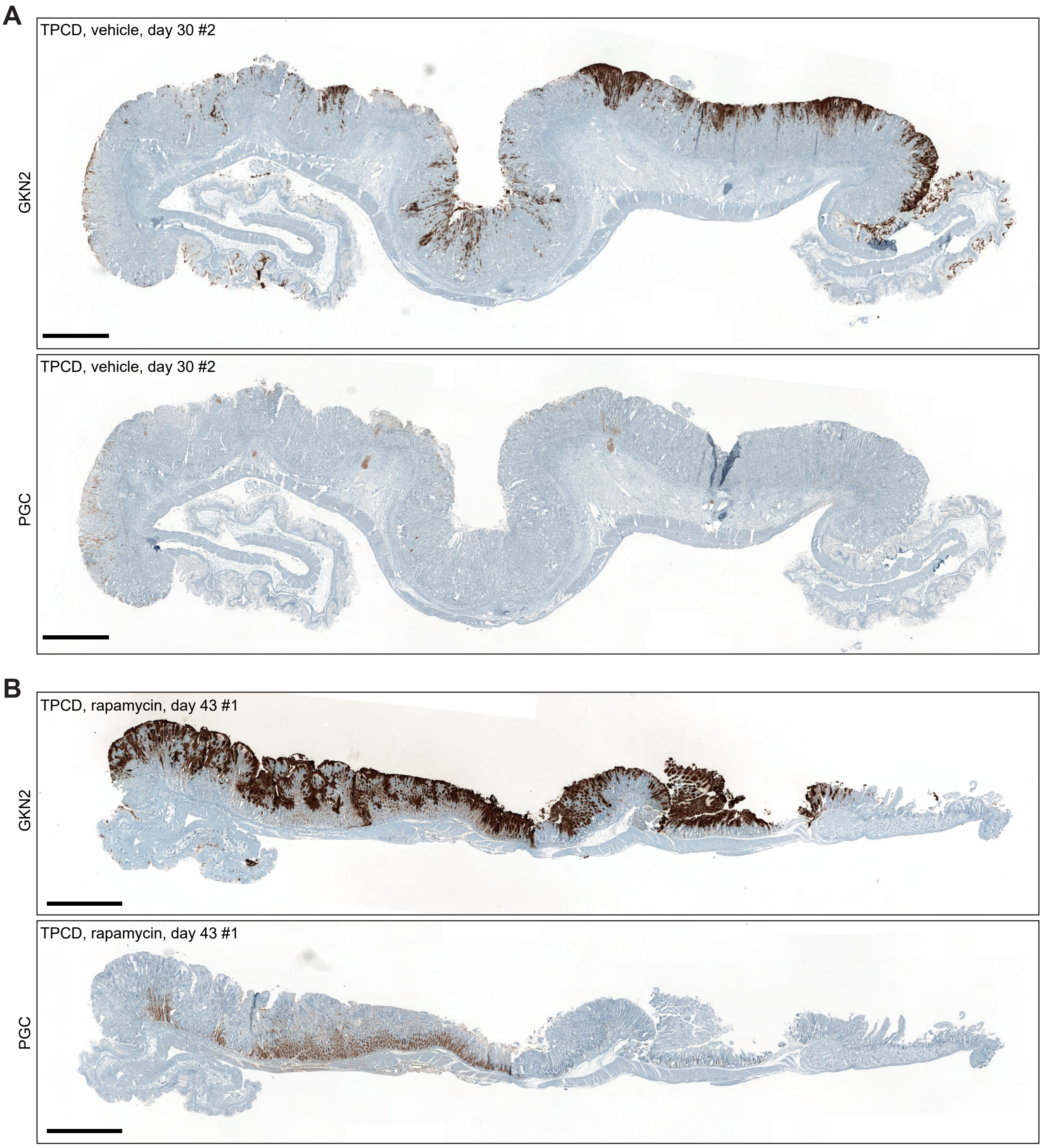

Figure S16. Rapamycin treatment rescues gastric differentiation in TPCD mice.

A-B, Representative IHC of GKN2 and PGC in TPCD stomach tissues treated with vehicle or rapamycin. Scale bar, 1 mm.

Figure S17. Combination of rapamycin and anti-PD1 suppresses the growth of TPCD allografts.

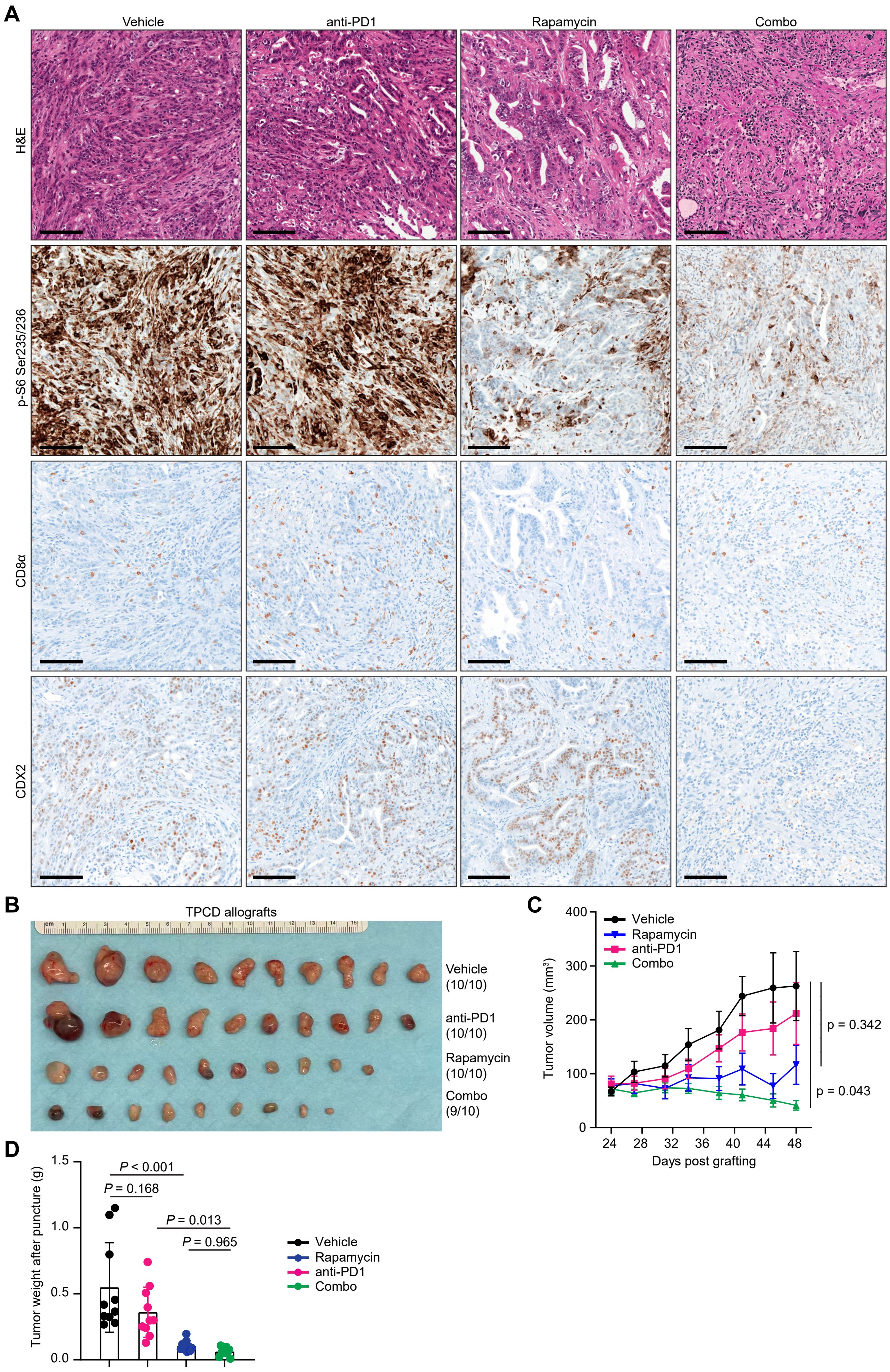

Figure S17. Combination of rapamycin and anti-PD1 suppresses the growth of TPCD allografts.

A, Representative H&E and IHC staining of p-S6 Ser235/236, CD8α, and CDX2 in grafted TPCD tumor tissues. Scale bar, 100 μm.

B-C, Allografts and growth curves of TPCD tumors treated with rapamycin (5 mg/kg/day) and/or anti-PD1 (8 mg/kg, twice a week) in C57BL/6 mice (n=10 mice per condition). Treatment started 3.5 weeks after injection of cells into the mammary fat pad. Data are presented as mean ± SEM and analyzed using mixed-effects model (REML) without sphericity assumption, followed by Sidak's multiple comparisons test at endpoint.

D, Statistics of TPCD tumor weight in C57BL/6 mice. Data are presented as mean ± SD and analyzed using two-way ANOVA followed by Tukey's multiple comparisons test..
