# Supplementary material for: Loss of Kmt2c/d promotes gastric cancer and confers vulnerability to mTORC1 and PD-1 inhibition
Source: J Clin Invest. 2026 May 12;136(14):e194462. doi: 10.1172/JCI194462 (PMC13367975; doi:10.1172/JCI194462)

Uncropped gel for Figure S10E

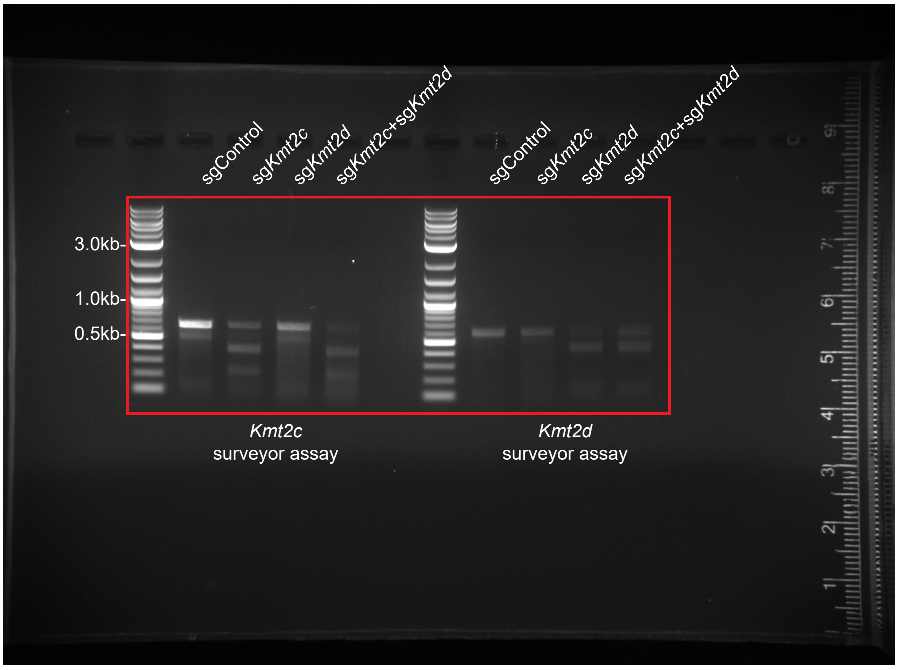

Uncropped gel for Figure S10F

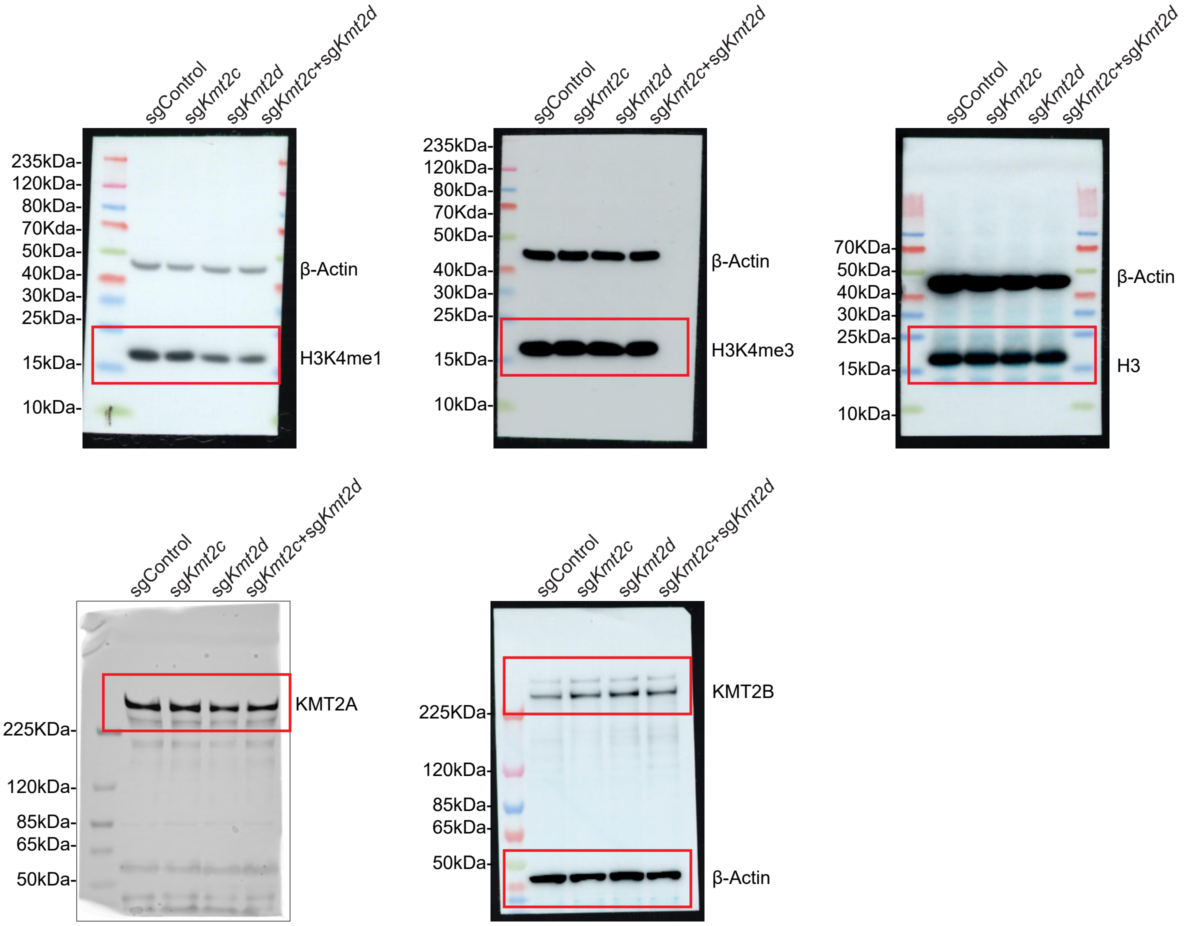

Uncropped gel for Figure S11B

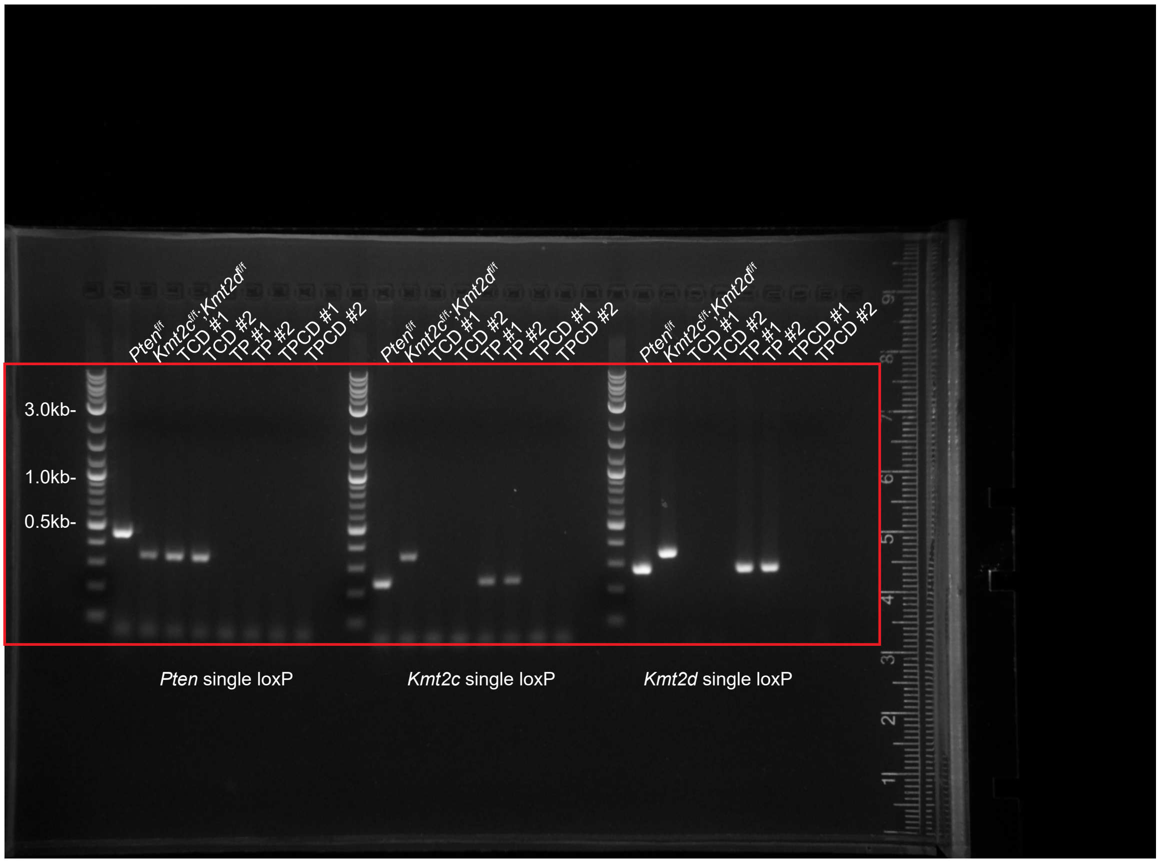

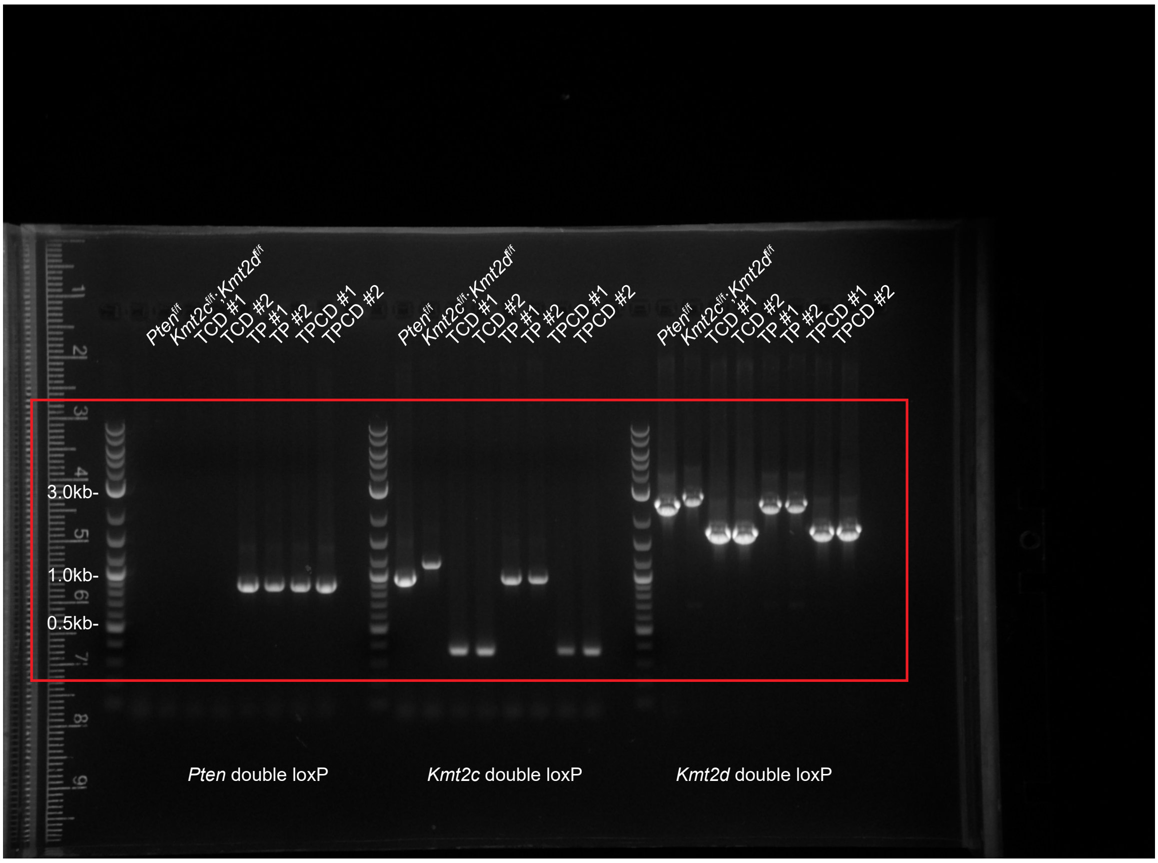

Uncropped gel for Figure S14B

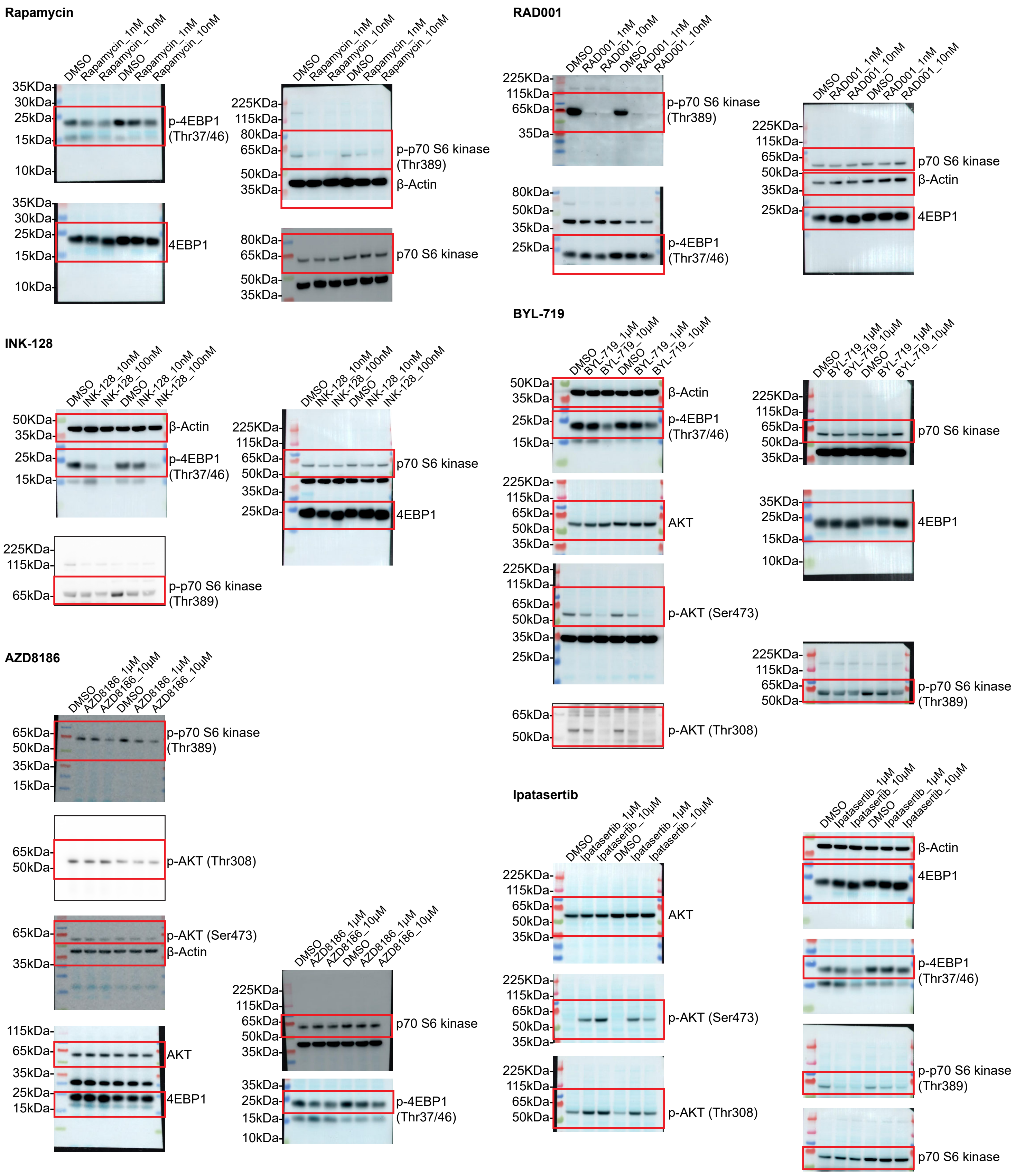

Supplement: Unedited blot and gel images [file jci-136-194462-s297.pdf]
